# Stereodynamics of Hemiacetal Formation in Pyridine‐3‐Carbaldehyde and Pyrimidine‐5‐Carbaldehyde Derivatives: Implications for the Soai Reaction

**DOI:** 10.1002/chir.70152

**Published:** 2026-10-03

**Authors:** Patrick Möhler, Alexander F. Siegle, Oliver Trapp

**Affiliations:** ^1^ Department of Chemistry Ludwig‐Maximilians‐University Munich Munich Germany

**Keywords:** asymmetric autocatalysis, chiral stationary phase, dynamic HPLC (DHPLC), Eyring analysis, hemiacetal formation, Soai reaction

## Abstract

The Soai reaction is a unique example of self‐amplifying asymmetric autocatalysis, in which a chiral zinc alkoxide not only promotes its own formation from pyridine‐3‐carbaldehydes and pyrimidine‐5‐carbaldehydes by alkylation with diisopropylzinc but also amplifies the enantiomeric excess of the added nonracemic alcohol. Both the asymmetric autocatalysis and the positive nonlinear effect have been attributed to equilibria involving the zinc alkoxide product, including dimerization and hemiacetalate formation with the substrate aldehyde. Hemiacetal formation is therefore of central importance for the efficiency of the autocatalytic cycle. Here, we report kinetic investigations of the dynamic hemiacetal formation of Soai aldehydes, showing a correlation of hemiacetal formation rates with autocatalytic reactivity. Hemiacetal formation with a series of alcohols was studied by ^1^H NMR spectroscopy and temperature‐dependent dynamic HPLC (DHPLC), providing mechanistic insight into the stereodynamics governing this key equilibrium. The Gibbs free activation energy Δ*G* ^ǂ^ for the formation of hemiacetals with isopropanol for pyridine‐3‐carbaldehydes is in the range between 94.6 and 95.5 kJ/mol and for pyrimidine‐5‐carbaldehydes it is between 85.9 and 88.4 kJ/mol.

## Graphical abstract

Kinetic studies of the hemiacetal formation of pyridine‐3‐carbaldehyde and pyrimidine‐5‐carbaldehyde derivatives with alcohols by ^1^H NMR and dynamic HPLC show a high correlation between hemiacetal formation and autocatalytic reactivity in Soai's asymmetric autocatalysis with Gibbs activation energies between 85.9 and 95.5 kJ/mol.

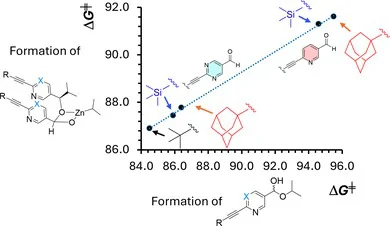

## 1 Introduction

Dynamic and stereochemically flexible catalyst systems and ligands offer the unique opportunity of controlling the handedness of a reaction product upon exposition to a stimulus [1, 2]. Catalyst systems have been developed that are able to sense the chirality of a chiral auxiliary [3, 4]. Recognition between a dynamic catalyst and a chiral auxiliary can activate or deactivate one of two possible active forms of the catalyst [5, 6, 7].

Dynamic supramolecular and covalent catalyst interactions and aggregations do not only enable asymmetric catalysis but also provide the possibility for nonlinear effects (NLEs) and self‐amplification [8, 9, 10, 11]. A well‐known example for an asymmetric reaction with a strong positive NLE is the alkylation of aromatic aldehydes with dialkylzinc reagents catalyzed by chiral β‐aminoalcohols [12, 13].

The nonlinearity of the asymmetric alkylation reaction originates from the aggregation of the chiral zinc alkoxides into hetero‐ and homochiral dimers. Due to a strong recognition of the mirror image, the heterochiral dimer is thermodynamically more stable than the homochiral dimer. The difference in stability leads to a higher proportion of the excess monomeric enantiomer from a small initial imbalance [14, 15].

Soai's autocatalysis [16] represents a unique example that combines a dynamic catalyst formation and a strong positive NLE. The substrate scope of the Soai reaction includes heterocyclic aldehydes, namely pyridine‐3‐ and pyrimidine‐5‐carbaldehydes. These are alkylated asymmetrically with diisopropyl zinc in the presence of catalytic amounts of the chiral alkoxide product. Like in Noyori's mechanism, the origin of the strong positive NLE is attributed to the reversible homochiral and heterochiral interactions of the enantiomeric zinc amino alkoxide catalysts. While the heterochiral Zn dimer is thermodynamically stable but practically catalytically inactive, the homochiral dimer readily dissociates into the active monomeric catalyst [17]. As proposed by our group, the active catalyst is a chiral hemiacetalate which dynamically forms from a substrate aldehyde and a chiral zinc alkoxide (Figure 1) [18]. The incorporation of the aldehyde into the catalytically active structure is reflected in a second order rate dependency in aldehyde concentration [18, 19, 20].

**FIGURE 1:**
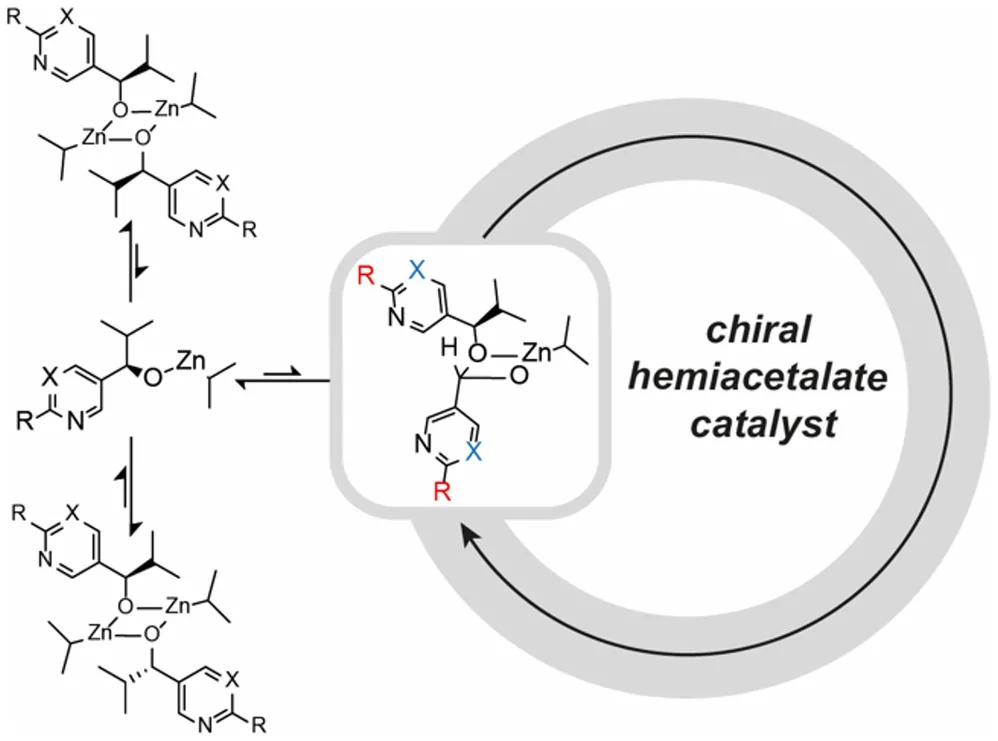
Dimerization and hemiacetalate formation equilibria of the autocatalytic zinc alcoholate during the Soai reaction [18, 19].

In the autocatalytic mechanism, hemiacetalate formation is the rate‐limiting step of the autocatalytic cycle of the Soai reaction [18, 19]. Therefore, hemiacetal‐formation rates should directly correlate with autocatalytic reactivity. In this work, we investigate the equilibrium between Soai aldehydes and the corresponding hemiacetals in the presence of different alcohols to establish a relationship between hemiacetal formation rates and autocatalytic reactivity.

## 2 Materials and Methods

### 2.1 General Procedures

All reactions involving the use of moisture and/or oxygen‐sensitive substances were carried out in glassware, previously dried with a heat gun under argon atmosphere, using standard Schlenk techniques. Syringes were used to transfer solvents or reagents and purged three times with argon prior to use.

### 2.2 Solvents and Reagents

All reagents were obtained from Sigma‐Aldrich, Germany, and abcr, Karlsruhe, Germany, and were used without further purification. The dry solvents toluene and THF were taken from the solvent purification system MB SPS‐800 and stored under argon.

### 2.3 Analytical Methods

NMR spectra were recorded on a Bruker AVIII HD400 and a Varian vnmrs spectrometer. Chemical shifts are reported as δ‐values in ppm referenced to the residual solvent peak [21]. For the characterization of the observed signal multiplicities, the following abbreviations were used: m (multiplet), s (singlet), d (doublet), t (triplet), p (pentet), dd (doublet of doublet), and dt (doublet of triplet).

High‐resolution mass spectrometry was performed using flow injection analysis (FIA/ESI) with a Surveyor MS pump at a flow rate of 100 μL/min with acetonitrile/water as the running agent. Each 1–10 μL sample solution was injected using an inline filter. EI spectra were recorded on Finnigan MAT 95Q or Finnigan MAT 90 instruments for electron impact ionization (EI). In situ mass spectrometric studies were performed using a Q Exactive Plus Orbitrap mass spectrometer (Thermo Scientific).

### 2.4 6‐((Trimethylsilyl)ethynyl)nicotinaldehyde (TMSPyr‐CHO)

This compound was prepared according to the literature [22]. 6‐Bromo‐pyridine‐3‐carboxaldehyde (1.86 g, 10.0 mmol, 1.00 equiv.), Pd (PPh_3_)_4_ (231 mg, 0.50 mmol, 5 mol%) and CuI (95.2 mg, 531 μmol, 5 mol%) were dissolved in degassed THF (25 mL). The mixture was cooled in an ice bath and *N,N*‐diisopropylethylamine (6.80 mL, 40.0 mmol, 4.00 equiv.) was added. After the mixture was stirred at 0°C for 5 min, trimethylsilylacetylene (1.52 mg, 11.0 mmol, 1.10 equiv.) was added dropwise. The ice bath was removed after 1 h and the mixture was stirred at room temperature for 6 h. Afterwards, the reaction mixture was filtered through Celite. The solvents were removed under reduced pressure. Purification of the crude material was achieved by sublimation (0.015 mbar, 65°C–70°C) to obtain TMSPyr‐CHO as a white crystalline solid (1.36 g, 6.70 mmol, 77%).

^1^H NMR (CDCl_3_, 400 MHz): δ [ppm] = 10.10 (d, J = 0.5 Hz, 1H), 9.02 (dd, J = 2.2, 0.9 Hz, 1H), 8.12 (dd, J = 8.1, 2.1 Hz, 1H), 7.60 (dt, J = 8.1, 0.8 Hz, 1H), 0.29 (s, 9H). ^13^C NMR (CDCl_3_, 400 MHz): δ [ppm] = 189.3, 152.2, 147.9, 135.5, 130.9, 127.6, 103.6, 100.1, 0.5. HR‐MS (ESI): *m/z* calc. for C_12_H_13_NOSi [M + H]^+^: 204.0839; found: 204.0838.

### 2.5 5‐Bromo‐2‐((trimethylsilyl)ethynyl)pyrimidine

The synthesis is a modification of a reported procedure [23]. Diisopropylamine (7.73 mL, 54.8 mmol, 4.00 equiv.), CuI (156 mg, 0.82 mmol, 6 mol%), Pd (PPh_3_)_4_ (475 mg, 0.41 mmol, 3 mol%), and 5‐bromo‐2‐iodo‐pyrimidine 6 (3.9 g, 13.7 mmol, 1.00 equiv.) were dissolved in degassed THF (60 mL). The mixture was cooled to 0°C before dropwise adding trimethylsilylacetylene (2.10 mL, 15.1 mmol, 1.10 equiv.). After 30 min, the ice bath was removed, and the reaction mixture was stirred at room temperature for 15 h. The reaction mixture was diluted with diethyl ether (100 mL), filtered through Celite and washed with diethyl ether (100 mL). The solvent was removed under reduced pressure, and the crude product was purified via flash column chromatography (cyclohexane/ethyl acetate, 33/1) to give 5‐bromo‐2‐((trimethylsilyl)ethynyl)pyrimidine as a white solid (2.94 g, 11.5 mmol, 84%).

^1^H NMR (CDCl_3_, 400 MHz): δ [ppm] = 8.75 (s, 2H), 0.29 (s, 9H). ^13^C NMR (CDCl_3_, 151 MHz): δ [ppm] = 157.9, 150.2, 119.5, 101.3, 96.2, −0.5. HR‐MS (EI): *m/z* calc. for C_9_H_11_BrN_2_Si: 253.9875, found: 253.9869.

### 2.6 2‐(Ethynyltrimethyllsilane)‐Pyrimidine‐5‐Carbaldehyde (TMSPym‐CHO)

This synthesis is a modification of a reported procedure [24]. 5‐Bromo‐2‐((trimethylsilyl)ethynyl)pyrimidine (1.02 g, 4.00 mmol, 1.00 equiv.) was dissolved in dry THF (40 mL). The solution was cooled to −78°C and ethyl formate (0.48 mL, 6.00 mmol, 1.50 equiv.) was added. After 5 min, *n*BuLi (1.90 M in hexane, 3.16 mL, 6.00 mmol, 1.50 equiv.) was added dropwise. The mixture was stirred for 5 min and subsequently quenched by the addition of concentrated acetic acid (0.35 mL). The mixture was warmed to room temperature and saturated Na_2_CO_3_ solution (20 mL) was added. The aqueous and organic layers were separated, and the aqueous layer was extracted with CH_2_Cl_2_ (3 × 25 mL). The solvents were removed under reduced pressure, and the obtained crude material was purified via column chromatography (cyclohexane/ethyl acetate, 10/1). TMSPym‐CHO was obtained as a beige solid (363 mg, 1.76 mmol, 45%).

^1^H NMR (CDCl_3_, 400 MHz): δ [ppm] = 10.14 (s, 1H), 9.14 (s, 2H), 0.32 (s, 9H). ^13^C NMR (CDCl_3_, 151 MHz): δ [ppm] = 188.3, 158.4, 155.4, 126.7, 102.0, 100.0, −0.5. HR‐MS (EI): *m/z* calc. for C_10_H_12_ON_2_Si: 204.0719, found: 204.0718.

### 2.7 Ethynyl Adamantane

An LDA solution (1.00 M in THF, 15.0 mL, 15.0 mmol, 1.00 equiv.) was cooled to −78°C and a solution of adamantyl methyl ketone (2.67 g, 15.0 mmol, 1.00 equiv.) in dry THF (7 mL) was added dropwise. After stirring for 1 h, chloro diethyl phosphate (2.16 mL, 15.0 mmol, 1.00 equiv.) was added slowly and the reaction mixture was allowed to warm to room temperature. After stirring for 3 h, this mixture was added dropwise to a second LDA solution (1.00 M in THF, 30.0 mL, 30.0 mmol, 2.00 equiv.) at −78°C. The solution was stirred at room temperature for 15 h. The reaction was quenched by the addition of water (50 mL). After separating the organic and aqueous layers, the aqueous layer was extracted with pentane (3 × 50 mL). The combined organic extracts were washed with ice‐cold aqueous HCl (1 M, 2 × 70 mL) and saturated NaHCO_3_ solution (100 mL). After drying over Na_2_SO_4_ and filtration through Celite, the solvent was removed under vacuum. Purification was performed by flash column chromatography (cyclohexane/ethyl acetate, 10/1). Ethynyl adamantane was obtained as a white solid (1.97 g, 12.3 mmol, 82%) [25].

^1^H NMR (CDCl_3_, 400 MHz): δ [ppm] = 2.10 (s, 1H), 1.96 (t, J = 3.2 Hz, 3H), 1.89 (d, J = 2.9 Hz, 6H), 1.69 (t, J = 3.2 Hz, 6H). ^13^C NMR (CDCl_3_, 151 MHz): δ [ppm] = 93.2, 66.7, 42.8, 36.4, 29.5, 28.0. HR‐MS (EI): *m/z* calc. for C_12_H_16_: 160.1252, found: 160.1245.

### 2.8 6‐((Adamantan‐1‐yl)Ethynyl)Nicotinaldehyde (AdPyr‐CHO)

This synthesis was performed as described previously [19, 25]. Ethynyl adamantane (1.06 g, 6.60 mmol, 1.10 equiv.) was dissolved in degassed THF (5 mL). In a separate Schlenk flask, 6‐bromonicotinaldehyde (1.12 g, 6.00 mmol, 1.00 equiv.), CuI (57.1 mg, 300 μmol, 5 mol%), and Pd (PPh_3_)_4_ (13.0 mg, 120 μmol, 0.02 equiv.) were dissolved in dry THF (50 mL). Diisopropylamine (3.20 mL, 24.0 mmol, 4.00 equiv.) was added and the mixture was degassed by freeze‐pump thaw (liquid N_2_, three times). The mixture was cooled to 0°C, and the ethynyl adamantane solution was added. After stirring at room temperature for 24 h, the reaction mixture was filtered through Celite. After removal of the solvent under reduced pressure, the crude material was purified via column chromatography (cyclohexane/ethyl acetate, 10/1) to yield compound AdPyr‐CHO as a white solid (1.32 g, 4.98 mmol, 83%).

^1^H NMR (CDCl_3_, 400 MHz): δ [ppm] = 10.08 (s, 1H), 8.99 (d, J = 2.2 Hz, 1H), 8.10 (dd, J = 8.1, 2.1 Hz, 1H), 7.52 (d, J = 8.1 Hz, 1H), 2.01 (d, J = 1.3 Hz, 10H), 1.73 (d, J = 2.4 Hz, 6H). ^13^C NMR (CDCl_3_, 151 MHz): δ [ppm] = 197.4, 189.8, 151.9, 135.8, 129.5, 127.3, 77.3, 77.0, 76.7, 42.1, 36.2, 30.3, 27.7. HR‐MS (EI): *m/z* calc. for C_18_H_19_NO: 265.1467, found: 265.1462.

### 2.9 5‐Bromo‐2‐((adamantyl)ethynyl)pyrimidine

5‐Bromo‐2‐iodo‐pyrimidine (1.20 g, 4.23 mmol, 1.00 equiv.), CuI (32.2 mg, 0.17 mmol, 4 mol%), and Pd (PPh_3_)_4_ (97.7 mg, 84.5 μmol, 2 mol%) were dissolved in degassed THF (18 mL). To this mixture, diisopropylamine (2.39 mL, 16.9 mmol, 4.00 equiv.) and ethynyl adamantane (745 mg, 4.65 mmol, 1.10 equiv.) were added at 0°C. Afterwards, the reaction mixture was stirred at room temperature for 48 h, filtered through Celite and the solvents were removed under reduced pressure. Purification of the crude product was achieved via column chromatography (cyclohexane/ethyl acetate, 50/1) to yield the product as a white solid (1.32 g, 4.16 mmol, 90%).

^1^H NMR (CDCl_3_, 600 MHz): δ [ppm] = 8.72 (s, 2H), 2.01 (s, 9H), 1.72 (s, 6H). ^13^C NMR (CDCl_3_, 151 MHz): δ [ppm] = 157.9, 151.4, 118.6, 99.3, 78.3, 42.0, 36.3, 30.2, 27.8. HR‐MS (EI): *m/z* calc. for C_16_H_17_N_2_Br: 316.0575, found: 316.0563.

### 2.10 2‐(Ethynyl‐adamantyl)‐Pyrimidine‐5‐Carbaldehyde (AdPym‐CHO)

This synthesis is a modification of a reported procedure [23]. AdPym‐Br (700 mg, 2.21 mmol, 1.00 equiv.) was dissolved in dry THF (20 mL). The solution was cooled to −78°C and ethyl formate (0.27 mL, 3.31 mmol, 1.50 equiv.) was added and the mixture was stirred for 3 min. Afterwards, *n*BuLi (2.5 M in hexane, 1.32 mL, 3.31 mmol, 1.50 equiv.) was added over 10 min and the mixture was stirred at −78°C for an additional 15 min. The reaction was quenched by the dropwise addition of acetic acid (0.19 mL, 3.31 mmol, 1.50 equiv.) and saturated Na_2_CO_3_ solution (20 mL) was added. The organic and aqueous layers were separated, and the aqueous layer was extracted with CH_2_Cl_2_ (3 × 20 mL). The organic phase was dried over Na_2_SO_4_, filtered off and the solvent was removed under vacuum. Purification of the crude product was performed via column chromatography (cyclohexane/ethyl acetate, 20/1). AdPym‐CHO was obtained as a white solid (295 mg, 1.11 mmol, 51%).

^1^H NMR (CDCl_3_, 600 MHz): δ [ppm] = 10.12 (s, 1H), 9.10 (s, 2H), 2.07–2.00 (s, 9H), 1.74 (d, J = 2.9 Hz, 6H). ^13^C NMR (CDCl_3_, 151 MHz): δ [ppm] = 188.6, 158.6, 156.8, 126.5, 103.1, 79.7, 42.1, 36.4, 30.6, 27.9, 27.2. HR‐MS (EI): *m*/*z* calc. for C_17_H_18_N_2_O: 266.1419, found: 266.1415.

Dynamic HPLC (DHPLC) measurements were performed on an Agilent 1200 Infinity HPLC equipped with a binary pump, an autosampler (Agilent HiP+), a thermostated column oven and a photodiode array detector (DAD). All operations were controlled by the Agilent Chemstation software. The separations were performed on immobilized chiral stationary phases (Chiralpak IA, Chiralpak IB, Chiralpak IC from Chiral Technologies, Illkirch, France) using *n*‐hexane/iPrOH = 60/40 (*v/v*) as mobile phase at a flow rate of 1.0 mL/min. The investigations were conducted with the similar mobile phase to exclude solvent‐dependent influences on activation parameters. The columns were preheated for at least 30 min prior to the measurements at a specific temperature. It should be noted that the temperature‐dependent measurements were conducted as the hemiacetal formation was equilibrated at room temperature. The on‐column equilibrium between the aldehyde and the hemiacetal of the pyridine system TMSPyr‐CHO, was analyzed on a Chiralpak IC column (250 mm, ID 4.6 mm, particle size: 5 μm) from Chiral Technologies. The on‐column equilibrium between the aldehyde and the hemiacetal of the pyridine system AdPyr‐CHO, was monitored on a Chiralpak IA column (250 mm, ID 4.6 mm, particle size: 5 μm) from Chiral Technologies. The on‐column equilibria between the aldehyde and the hemiacetal of the pyrimidine systems TMSPym‐CHO and AdPym‐CHO, were monitored on a Chiralpak IB column (250 mm, ID 4.6 mm, particle size: 5 μm) from Chiral Technologies. The equilibrium and the dynamics were monitored between 20°C and 70°C.

## 3 Results and Discussion

The aldehyde–hemiacetal equilibrium was investigated for several Soai aldehydes by NMR and DHPLC. For our experiments, we chose aldehydes that have shown high efficiency in Soai's asymmetric autocatalysis [16, 22, 25, 26, 27]. The actual transient hemiacetalate catalyst is highly reactive and only formed to a small extent under reaction conditions; therefore, thermodynamic and kinetic insights on hemiacetal formation were obtained in alcoholic solvents and other alcoholic model systems.

### 3.1 NMR Experiments

The extent of hemiacetal formation can easily be quantified by ^1^H NMR spectroscopy with deuterated alcohols as solvents. In our experiments, the Soai aldehydes were dissolved in MeOD‐*d*_4_ and iPrOD‐*d*_8_ and the resulting equilibrium between the different species was monitored for 60 h (Scheme 1, cf. Figures S1.1.1–S1.2.4 in the Supporting Information).

**SCHEME 1:**
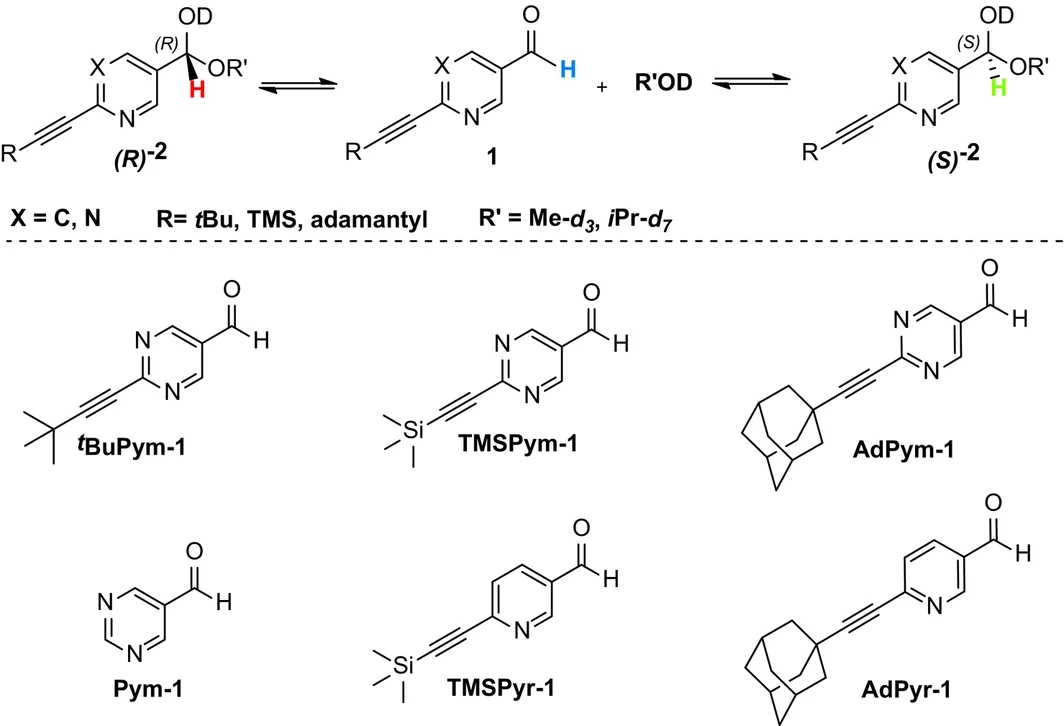
Investigated hemiacetal equilibria of pyridine‐3‐ and pyrimidine‐5‐carbaldehydes in deuterated alcohols MeOD‐*d*_4_ and iPrOD‐*d*_8_.

In some cases, it took several hours or days for the equilibrium between aldehyde and hemiacetal to be reached. The hemiacetal yield was calculated from the ratio of the aldehyde signal and the emerging characteristic hemiacetal proton between 5.5 and 6.0 ppm. The results are summarized in Table 1.

**TABLE 1:** ^1^H NMR hemiacetal yields of different aromatic aldehydes after 60 h.

Entry	Aldehyde	Alcohol	Hemiacetal yield
1	Benzaldehyde	MeOD‐*d*_4_	9% [28]
2	Pym‐1	MeOD‐*d*_4_	95% [28]
3	tBuPym‐1	MeOD‐*d*_4_	95% [28]
4	TMSPym‐1	MeOD‐*d*_4_	96%
5	AdPym‐1	MeOD‐*d*_4_	95%
6	TMSPyr‐1	MeOD‐*d*_4_	79%
7	AdPyr‐1	MeOD‐*d*_4_	71%
8	TMSPym‐1	iPrOD‐*d*_8_	87%
9	AdPym‐1	iPrOD‐*d*_8_	67%
10	TMSPyr‐1	iPrOD‐*d*_8_	32%
11	AdPyr‐1	iPrOD‐*d*_8_	19%

In both solvents, in MeOD‐*d*_4_ and in iPrOD‐*d*_8_, the amount of hemiacetal formed is significantly higher for pyrimidine aldehydes compared to their pyridine analogs. Therefore, the electron‐withdrawing effect of the second nitrogen atom in the heterocyclic ring has a strong impact on the extent of the hemiacetal formation. In MeOD‐*d*_4_ the equilibrium between aldehyde and hemiacetal is shifted almost completely towards the latter for the pyrimidine derivatives with 96% (TMSPym‐1) and 95% (AdPym‐1, *t*BuPym‐1) hemiacetal yield.

With hemiacetal yields of 79% (TMSPyr‐1) and 71% (AdPyr‐1), the yields are significantly lower for the pyridine derivatives, but the equilibria are still shifted towards the hemiacetals. In this context, it should be mentioned that benzaldehyde in MeOD‐*d*_4_ forms the corresponding hemiacetal in only 9% yield [18]. These observations imply the necessity of at least one heterocyclic nitrogen atom for efficient hemiacetal formation.

The results also show the influence of the alcohol's steric demand on hemiacetal formation. For all tested aldehydes, the hemiacetal yield in iPrOD‐*d*_8_ is considerably lower than in MeOD‐*d*_4_. In iPrOD‐*d*_8_, the equilibrium is shifted towards the hemiacetal for both pyrimidines with 87% (TMSPym‐1) and 67% (AdPym‐1) yield, while the pyridine derivatives were found to form the corresponding hemiacetal to a significantly lower extent with hemiacetal yields of 32% (TMSPyr‐1) and 19% (AdPyr‐1). For the adamantyl‐substituted pyridine aldehyde AdPyr‐1, the hemiacetal yield decreased by 52% when the solvent was changed from MeOD‐*d*_4_ to iPrOD‐*d*_8_.

These structural influences on aldehyde and alcohol imply that the sterically demanding alcohol autocatalyst will form the catalytically active hemiacetal to a minor extent under reaction conditions, especially when the alcohol concentration is low at early stages of the reaction.

Therefore, the ability of the substrate aldehyde to form hemiacetals fast and efficiently is crucial for the autocatalysis to proceed.

Furthermore, it was observed that the TMS‐substituted aldehydes show increased hemiacetal yields compared to the adamantyl‐substituted derivatives. However, the alkyne substituent exhibits a minor influence on hemiacetal formation compared to the influence of the aromatic core.

Structural information on the diastereomeric hemiacetal catalyst in the Soai reaction was obtained by monitoring the hemiacetal formation of AdPym‐1 and 2‐methyl‐1‐phenyl‐1‐propanol (MPP). MPP is structurally related to the autocatalytic Soai alcohols and was therefore used as a model system. After dissolving the aldehyde and equimolar amounts of MPP in toluene‐*d*_8_, two signals emerged in the hemiacetal region of the ^1^H NMR spectrum at 5.1 ppm and 5.3 ppm in a ratio of 5 to 1. By ^1^H‐^13^C HSQC measurements, the two signals could be assigned to carbon atoms with shifts of 90 and 94 ppm (Figure 2). The data suggest the formation of hemiacetal diastereomers with a preferential formation of one pair of enantiomers, which is in agreement with mechanistic proposals from our group [18, 19]. According to the proposal, the homochiral enantiomers with either *R,R‐* or *S,S*‐configuration are favored compared to their heterochiral *R,S*‐ or *S,R*‐configured isomers.

**FIGURE 2:**
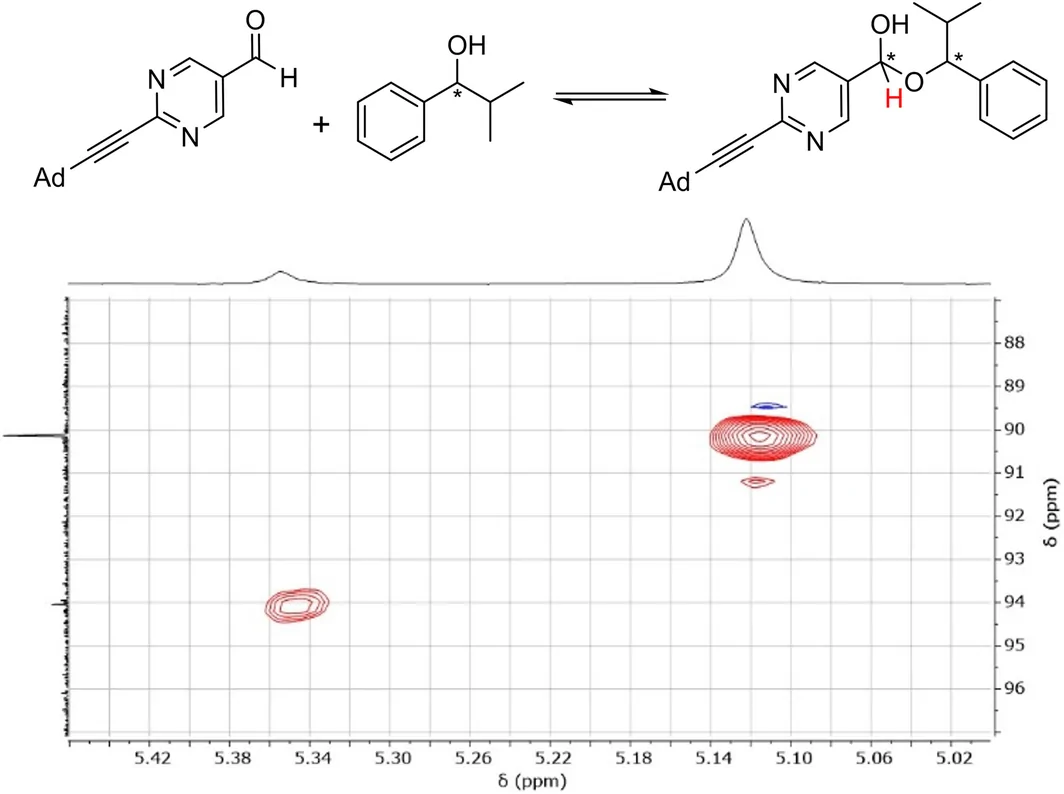
^1^H‐^13^C HSQC spectrum of hemiacetal diastereomers from AdPym‐1 and 2‐methyl‐1‐phenyl‐1‐propanol in toluene‐*d*_8_.

While the presented NMR data gives valuable insights into the position of the investigated equilibria, it does not provide kinetic insights into this process.

### 3.2 Temperature‐Dependent DHPLC

DHPLC represents a powerful tool for kinetic evaluation of dynamic on‐column processes such as the interconversion of stereoisomers [29, 30, 31, 32, 33, 34, 35, 36]. For instance, the method has been widely used for the investigation of configurational stabilities of chiral compounds. In previous studies, the dynamic on‐column hemiacetal formation of aldehydes with iPrOH in the mobile phase was investigated with DHPLC [18]. The observation of hemiacetals by chromatographic techniques enables investigating their rate of formation. Although the Soai reaction is performed under different aprotic conditions it can be expected that the rate of hemiacetal formation with iPrOH correlates with the hemiacetal formation rate of the Soai aldehydes and the product zinc alcoholates. Therefore, we assume that the hemiacetal formation rate with isopropanol can be correlated with autocatalytic reactivity.

For our experiments, the analyte aldehyde was dissolved in a *n*‐hexane/iPrOH solvent mixture and the DHPLC analysis was carried out after the aldehyde–hemiacetal ratio was found to be steady (Figure 3A, experimental conditions are described in detail in the Supporting Information). Especially for pyridine‐based aldehydes, the equilibration took several hours. The resulting equilibrium was subsequently analyzed on a tempered chiral column in a temperature range between 20°C and 70°C. Chromatograms from HPLC separations of pyridine‐ and pyrimidine‐carbaldehydes with iPrOH in the mobile phase show a second peak that is connected to the aldehyde peak by a plateau. This additional peak can be assigned to hemiacetals as confirmed by HPLC‐MS. When using a suitable chiral stationary phase, the additional peak even separates into two peaks, which indicates the formation of hemiacetal enantiomers (Figure 3B). It should be noted that this on‐column equilibrium can be prevented by using other nonalcoholic solvents such as THF/*n*‐hexane as mobile phase. Here, the chromatographic behavior of the pyridine‐based aldehyde TMSPyr‐1 and pyrimidine‐based system TMSPym‐1 are discussed exemplarily to highlight the pronounced influence of the heterocycle on the equilibrium between the aldehyde and the hemiacetal and the kinetics of this transformation.

**FIGURE 3:**
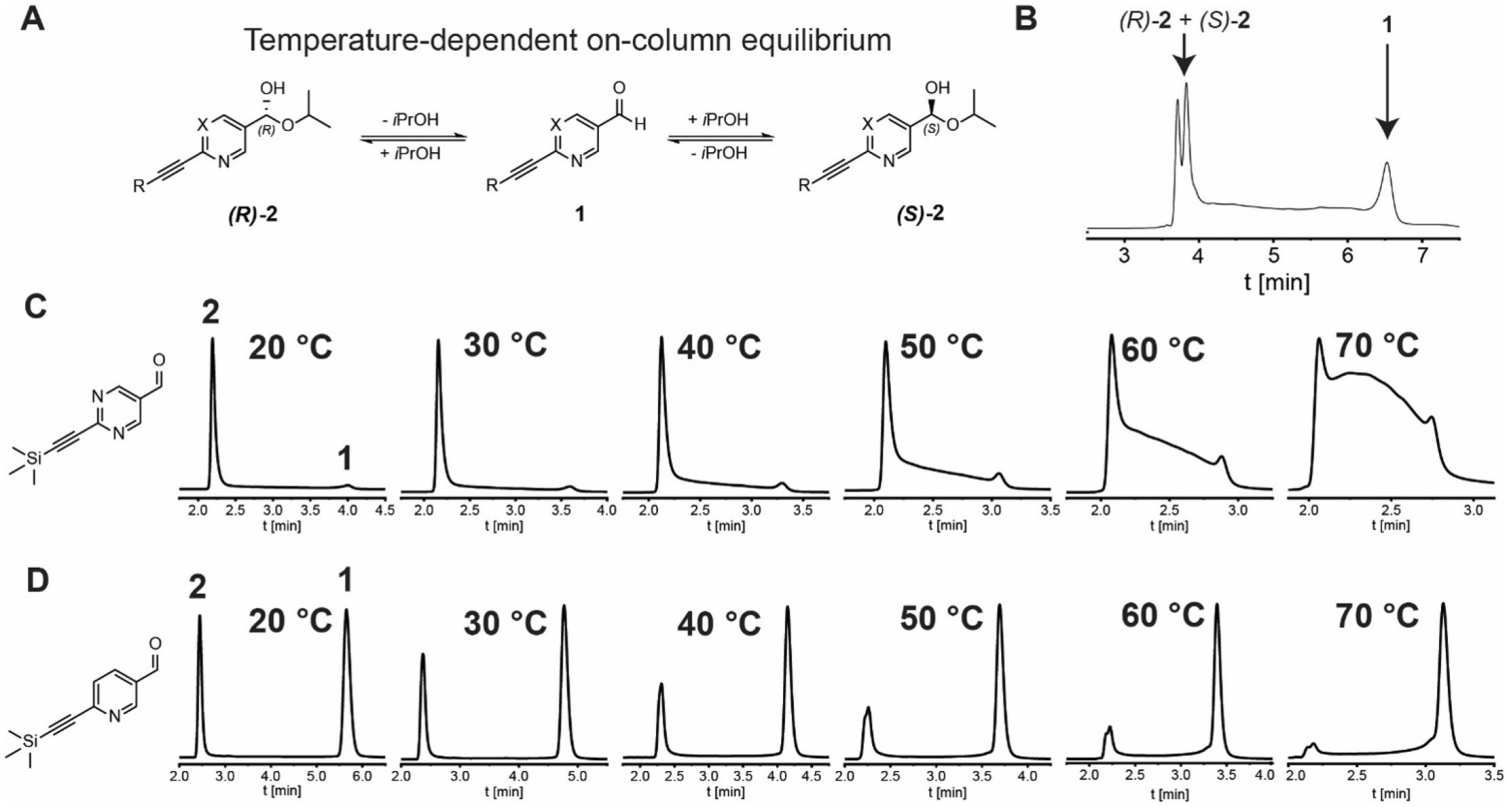
(A) Investigated temperature‐dependent on‐column equilibrium of different pyridine‐3‐ and pyrimidine‐5‐carbaldehydes and the corresponding chiral hemiacetals with iPrOH in the mobile phase. (B) Exemplary DHPLC chromatogram. The separation of hemiacetal enantiomers and a plateau formation between hemiacetal and aldehyde peaks was observed. (C) Dynamic chromatograms for the pyrimidine‐based aldehyde TMSPym‐1 (Chiralpak IB, *n*‐hexane/iPrOH = 60/40, 1 mL/min). (D) Dynamic chromatograms for the pyridine‐based aldehyde TMSPyr‐1 (Chiralpak IC, *n*‐hexane/iPrOH = 60/40, 1 mL/min) between 20°C and 70°C.

During the NMR experiments, significant differences were observed in the elution profiles of pyridine‐3‐carbaldehydes compared to their pyrimidine‐based analogs (Figure 3C,D). At 20°C, the temperature‐sensitive equilibrium is shifted almost entirely towards the hemiacetal for pyrimidine‐based systems (Figure 3C). Here, the aldehyde peak as well as the connecting plateau are barely visible. This observation is consistent with the NMR data, where the TMSPym‐1 system exhibited an 87% hemiacetal population in iPrOD‐*d*_8_ at room temperature (Table 1). In contrast, the profile of the pyridine‐based TMSPyr‐1 system at 20°C shows that the equilibrium is slightly shifted towards the aldehyde, which again matches a 32% hemiacetal fraction for this compound in iPrOD‐*d*_8_. With increasing temperatures, it is observed that the proportion of hemiacetal significantly decreases in favor of the aldehyde. For the pyridine‐based systems TMSPyr and AdPyr the hemiacetal form is almost completely absent at temperatures above 60°C. In comparison, the hemiacetal remains the dominating form of pyrimidine‐based systems at temperatures above 60°C.

This difference in temperature dependence provides an explanation for the inverse temperature effect of the Soai reaction, which proceeds faster at lower temperatures due to preferential formation of the hemiacetalate catalyst [20, 37]. However, the temperature sensitivity of the reaction kinetics should be even more pronounced for pyridine‐based systems since the hemiacetal formation is significantly more suppressed at higher temperatures.

Furthermore, important information about the interconversion rates can be derived from the plateau between the peaks. In general, the plateau tends to increase in height with increasing temperatures. This indicates that the interconversion rate between the different species increases with temperature. While this effect is negligible for pyridine‐based systems as the plateau height is low even at high temperatures, the differences are more prominent for the pyrimidine‐based systems. These differences imply that the processes of hemiacetal formation and decomposition are significantly slower for pyridine‐carbaldehydes.

The electron‐withdrawing effect of the second heterocyclic nitrogen atom increases the electrophilicity of the carbonyl carbon and appears to facilitate hemiacetal formation and decomposition both thermodynamically and kinetically. Consequently, this behavior explains the enhanced autocatalytic activity of pyrimidine‐based aldehyde substrates as the hemiacetalate catalyst can be formed not only to a greater extent but significantly faster compared to their pyridine analogs.

A quantitative kinetic analysis of the obtained chromatograms was performed by using the unified equation of dynamic chromatography implemented into the software DCXplorer [38, 39]. This allows the calculation of the apparent rate constants *k* of the dynamic on‐column hemiacetal formation and decomposition by mathematical separation of the dynamic profiles into noninterconverting peaks. The chromatographic parameters that are graphically analyzed by the DCXplorer software are the plateau height *h*_p_ between the two peaks, the retention times *t*_R_ of both peaks as well as the peak width *w*_h_. Due to the large excess of iPrOH in the mobile phase, the reaction follows pseudo‐first‐order kinetics.

From the obtained *k* values, the Gibbs activation energies Δ*G* ^╪^(*T*) were calculated using the Eyring equation. The activation enthalpy Δ*H* ^╪^ and the activation entropy Δ*S* ^╪^ for this process were obtained by linear regression analysis from the slope and the intercept of the Eyring plot (ln *k*/*T* vs. 1/*T*; Figure 4C). The results of the kinetic evaluation of the DHPLC measurements are summarized in Table 2.

**FIGURE 4:**
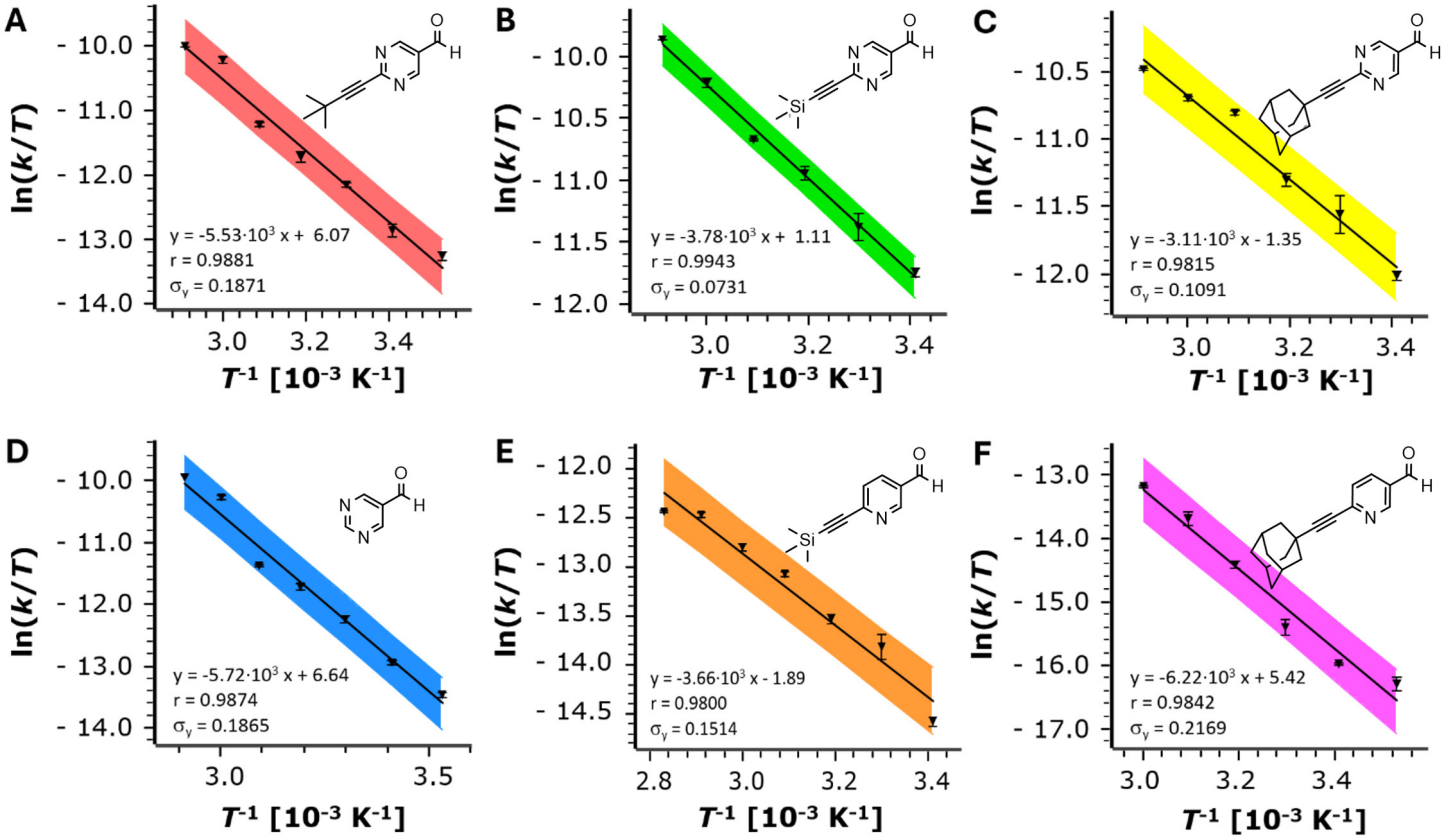
Eyring analysis (ln *k*/*T* vs. 1/*T*). Eyring analysis for the DHPLC measurements of the dynamic hemiacetal formation of six aldehydes with isopropanol. The activation enthalpy Δ*H* ^╪^ and the activation entropy Δ*S* ^╪^ were determined from the slope and the intercept of the Eyring plot. Each measurement was performed as a triplicate. The linear equation, the correlation coefficient *r* and the residual standard deviation *σ*_*y*_ obtained by regression analysis are depicted in the Eyring plots. Deviations of the activation parameters Δ*H* ^╪^ and Δ*S* ^╪^, have been calculated by analysis of the confidence interval of the linear regression with a level of confidence of 95%.

**TABLE 2:** Kinetic parameters obtained by Eyring analysis.

Entry	Aldehyde	Δ*G* ^ǂ^_298K_ [kJ/mol]	Δ*H* ^ǂ^ [kJ/mol]	Δ*S* ^ǂ^ [J/(K⋅mol)]
1	TMSPyr‐1	94.6 ± 0.1	43.0 ± 1.4	−173 ± 58
2	AdPyr‐1	95.5 ± 0.2	48.9 ± 2.4	−156 ± 25
3	Pym‐1	88.4 ± 0.1	47.8 ± 1.9	−136 ± 15
4	TMSPym‐1	85.9 ± 0.1	31.5 ± 0.8	−182 ± 52
5	AdPym‐1	86.4 ± 0.1	25.8 ± 0.6	−203 ± 31
6	*t*BuPym‐1	84.5 ± 0.2	26.3 ± 0.2	−195 ± 34

The results show that the activation energies Δ*G* ^ǂ^_298K_ for hemiacetal formation are 7–10 kJ/mol lower for pyrimidine‐5‐carbaldehydes (Figure 4A–D) compared to pyridine‐3‐carbaldehydes (Figure 4E and F). The acetylene substituent in 2‐position of pyrimidine‐5‐carbaldehydes is also found to slightly lower the activation barrier of hemiacetal formation about 1–2 kJ/mol compared to the unsubstituted system.

The group on the acetylene substituent (TMS, adamantyl, *t*Bu), however, has a comparatively minor influence and differences of <1 kJ/mol in Δ*G* ^ǂ^_298K_ are observed for these systems. The results from the kinetic evaluation by DHPLC therefore show that pyrimidine‐based aldehydes, which exhibit higher autocatalytic activity, show a lower Gibbs activation energy Δ*G* ^ǂ^_298K_ for hemiacetal formation.

A further trend is seen in the activation enthalpy Δ*H* ^*ǂ*^ values. Here, the values are significantly lower for the acetylene‐substituted pyrimidines (25.8–31.5 kJ/mol), suggesting a lower enthalpic barrier for hemiacetal formation.

A similar trend is represented in the activation entropy Δ*S* ^ǂ^, where it is suggested that the hemiacetal formation is entropically less favored for the substituted pyridines (−182 – (−203) J/(K⋅mol)). These values imply a highly ordered transition state during hemiacetal formation. The correlation of the Gibbs free activation energy Δ*G* ^ǂ^_298K_ for the reaction of iPrOH and pyridine‐3‐ and pyrimidine‐5‐carbaldehydes shows a very high linearity with the Δ*G* ^ǂ^_298K_ hemiacetal formation in Soai's asymmetric autocatalysis obtained by reaction network analysis [18, 19] (Figure 5, correlation coefficient *r* = 0.9989). In addition, it also clearly shows the correlation in reactivity for the pyridine‐3‐ and pyrimidine‐5‐carbaldehyde derivatives.

**FIGURE 5:**
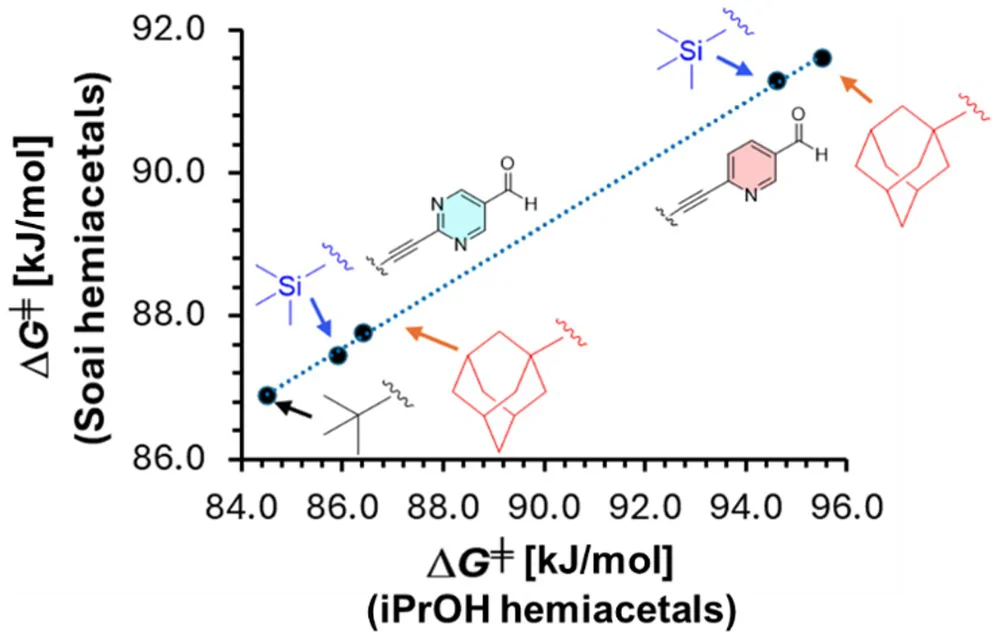
Correlation of the Gibbs free activation energies Δ*G* ^ǂ^_298K_ of the formation of hemiacetals by reaction with iPrOH and pyridine‐3‐ and pyrimidine‐5‐carbaldehydes, determined by DHPLC, and the formation of hemiacetals formed in situ in Soai's asymmetric autocatalysis obtained by reaction network analysis [18, 19]. The correlation coefficient *r* of the linear regression is 0.9989.

## 4 Conclusion

In this work, we have investigated the dynamic hemiacetal formation of several Soai aldehydes in methanol and isopropanol by ^1^H NMR spectroscopy and DHPLC. As a result, the different behaviors of pyridine‐3‐ and pyrimidine‐5‐carbaldehydes were demonstrated. Pyrimidine‐5‐carbaldehydes form hemiacetals to a larger extent compared to their pyridine analogs. DHPLC experiments allowed the calculation of the hemiacetal formation and decomposition rate constants, which revealed a significantly lower Gibbs free activation energy Δ*G* ^ǂ^_298K_ for the pyrimidine‐5‐carbaldehydes, consistent with faster hemiacetal formation. Consequently, a direct correlation of kinetic parameters of hemiacetal formation and autocatalytic reactivity during the Soai reaction is established here. We suggest that the determination of activation barriers for hemiacetal formation by DHPLC provides a basis for predicting reactivity during Soai's autocatalysis.

## Supporting information




**Figure S1.1.1** ^1^H NMR (400 MHz) of aldehyde TMSPyr in MeOD‐*d*_4_ after 60 h.
**Figure S1.1.2.** ^1^H NMR (400 MHz) of aldehyde AdPyr in MeOD‐d4 after 60 h.
**Figure S1.1.3.** ^1^H NMR (400 MHz) of aldehyde AdPym in MeOD‐d4 after 60 h.
**Figure S1.1.4.** ^1^H NMR (400 MHz) of aldehyde TMSPym in MeOD‐d4 after 60 h.
**Figure S1.2.1.** ^1^H NMR (400 MHz) of aldehyde TMSPyr in iPrOD‐d8 after 60 h.
**Figure S1.2.2.** ^1^H NMR (400 MHz) of aldehyde AdPyr in iPrOD‐d8 after 60 h.
**Figure S1.2.3.** ^1^H NMR (400 MHz) of aldehyde TMSPym in iPrOD‐d8 after 60 h.
**Figure S1.2.4.** ^1^H NMR (400 MHz) of aldehyde AdPym in iPrOD‐d8 after 60 h.
**Figure S2.3.1.** DHPLC profile obtained from temperature‐dependent measurements of the pyridine‐based aldehyde TMSPyr‐CHO in a n‐hexane/iPrOH (60/40) mobile phase at 20°C.
**Figure S2.3.2.** DHPLC profile obtained from temperature‐dependent measurements of the pyridine‐based aldehyde TMSPyr‐CHO in a n‐hexane/iPrOH (60/40) mobile phase at 30°C.
**Figure S23.3.** DHPLC profile obtained from temperature‐dependent measurements of the pyridine‐based aldehyde TMSPyr‐CHO in a n‐hexane/iPrOH (60/40) mobile phase at 40°C.
**Figure S2.3.4.** DHPLC profile obtained from temperature‐dependent measurements of the pyridine‐based aldehyde TMSPyr‐CHO in a n‐hexane/iPrOH (60/40) mobile phase at 50°C.
**Figure S2.3.5.** DHPLC profile obtained from temperature‐dependent measurements of the pyridine‐based aldehyde TMSPyr‐CHO in a n‐hexane/iPrOH (60/40) mobile phase at 60°C.
**Figure S2.3.6.** DHPLC profile obtained from temperature‐dependent measurements of the pyridine‐based aldehyde TMSPyr‐CHO in a n‐hexane/iPrOH (60/40) mobile phase at 70°C.
**Figure S2.3.7.** DHPLC profile obtained from temperature‐dependent measurements of the pyridine‐based aldehyde TMSPyr‐CHO in a n‐hexane/iPrOH (60/40) mobile phase at 80°C.
**Table S2.3.1.** Summarized experimental chromatographic parameters of the triple DHPLC measurements of the formation of the hemiacetal of TMSPyr with 2‐propanol.
**Table S2.3.2.** Summarized kinetic data of the triple DHPLC measurements of the formation of the hemiacetal of TMSPyr with 2‐propanol using the unified equation of chromatography.
**Figure S2.3.8.** Eyring plot for the determination of the activation parameters ΔH, ΔS, and ΔG of the hemiacetal formation with isopropanol obtained from the DHPLC experiment with the Soai aldehyde TMSPyr‐CHO. The upper and lower curves represent the error bands of the linear regression with a level of confidence of 95%.
**Figure S2.4.1.** DHPLC profile obtained from temperature‐dependent measurements of the pyridine‐based aldehyde TMSPym‐CHO in a n‐hexane/iPrOH (60/40) mobile phase at 20°C.
**Figure S2.4.2.** DHPLC profile obtained from temperature‐dependent measurements of the pyridine‐based aldehyde TMSPym‐CHO in a n‐hexane/iPrOH (60/40) mobile phase at 30°C.
**Figure S2.4.3.** DHPLC profile obtained from temperature‐dependent measurements of the pyridine‐based aldehyde TMSPym‐CHO in a n‐hexane/iPrOH (60/40) mobile phase at 40°C.
**Figure S2.4.4.** DHPLC profile obtained from temperature‐dependent measurements of the pyridine‐based aldehyde TMSPym‐CHO in a n‐hexane/iPrOH (60/40) mobile phase at 50°C.
**Figure S2.4.5.** DHPLC profile obtained from temperature‐dependent measurements of the pyridine‐based aldehyde TMSPym‐CHO in a n‐hexane/iPrOH (60/40) mobile phase at 60°C.
**Figure S2.4.6.** DHPLC profile obtained from temperature‐dependent measurements of the pyridine‐based aldehyde TMSPym‐CHO in a n‐hexane/iPrOH (60/40) mobile phase at 70°C.
**Table S2.4.1.** Summarized experimental chromatographic parameters of the triple DHPLC measurements of the formation of the hemiacetal of TMSPym with 2‐propanol.
**Table S2.4.2.** Summarized kinetic data of the triple DHPLC measurements of the formation of the hemiacetal of TMSPym with 2‐propanol using the unified equation of chromatography.
**Figure S2.4.7.** Eyring plot for the determination of the activation parameters ΔH, ΔS, and ΔG of the hemiacetal formation with isopropanol obtained from the DHPLC experiment with the Soai aldehyde TMSPym‐CHO. The upper and lower curves represent the error bands of the linear regression with a level of confidence of 95%.
**Figure S2.5.1.** DHPLC profile obtained from temperature‐dependent measurements of the pyridine‐based aldehyde AdPyr‐CHO in a n‐hexane/iPrOH (60/40) mobile phase at 10°C.
**Figure S2.5.2.** DHPLC profile obtained from temperature‐dependent measurements of the pyridine‐based aldehyde AdPyr‐CHO in a n‐hexane/iPrOH (60/40) mobile phase at 20°C.
**Figure S2.5.3.** DHPLC profile obtained from temperature‐dependent measurements of the pyridine‐based aldehyde AdPyr‐CHO in a n‐hexane/iPrOH (60/40) mobile phase at 30°C.
**Figure S2.5.4.** DHPLC profile obtained from temperature‐dependent measurements of the pyridine‐based aldehyde AdPyr‐CHO in a n‐hexane/iPrOH (60/40) mobile phase at 40°C.
**Figure S2.5.5.** DHPLC profile obtained from temperature‐dependent measurements of the pyridine‐based aldehyde AdPyr‐CHO in a n‐hexane/iPrOH (60/40) mobile phase at 50°C.
**Figure S2.5.6.** DHPLC profile obtained from temperature‐dependent measurements of the pyridine‐based aldehyde AdPyr‐CHO in a n‐hexane/iPrOH (60/40) mobile phase at 60°C.
**Figure S2.5.7.** DHPLC profile obtained from temperature‐dependent measurements of the pyridine‐based aldehyde AdPyr‐CHO in a n‐hexane/iPrOH (60/40) mobile phase at 70°C.
**Table S2.5.1.** Summarized experimental chromatographic parameters of the triple DHPLC measurements of the formation of the hemiacetal of AdPyr with 2‐propanol.
**Table S2.5.2.** Summarized kinetic data of the triple DHPLC measurements of the formation of the hemiacetal of AdPyr with 2‐propanol using the unified equation of chromatography.
**Figure S2.5.8.** Eyring plot for the determination of the activation parameters ΔH, ΔS, and ΔG of the hemiacetal formation with isopropanol obtained from the DHPLC experiment with the Soai aldehyde AdPyr‐CHO. The upper and lower curves represent the error bands of the linear regression with a level of confidence of 95%.
**Figure S2.6.1.** DHPLC profile obtained from temperature‐dependent measurements of the pyridine‐based aldehyde AdPym‐CHO in a n‐hexane/iPrOH (60/40) mobile phase at 10°C.
**Figure S2.6.2.** DHPLC profile obtained from temperature‐dependent measurements of the pyridine‐based aldehyde AdPym‐CHO in a n‐hexane/iPrOH (60/40) mobile phase at 20°C.
**Figure S2.6.3.** DHPLC profile obtained from temperature‐dependent measurements of the pyridine‐based aldehyde AdPym‐CHO in a n‐hexane/iPrOH (60/40) mobile phase at 30°C.
**Figure S2.6.4.** DHPLC profile obtained from temperature‐dependent measurements of the pyridine‐based aldehyde AdPym‐CHO in a n‐hexane/iPrOH (60/40) mobile phase at 40°C.
**Figure S2.6.5.** DHPLC profile obtained from temperature‐dependent measurements of the pyridine‐based aldehyde AdPym‐CHO in a n‐hexane/iPrOH (60/40) mobile phase at 50°C.
**Figure S2.6.6.** DHPLC profile obtained from temperature‐dependent measurements of the pyridine‐based aldehyde AdPym‐CHO in a n‐hexane/iPrOH (60/40) mobile phase at 60°C.
**Table S2.6.1.** Summarized experimental chromatographic parameters of the triple DHPLC measurements of the formation of the hemiacetal of AdPym with 2‐propanol.
**Table S2.6.2.** Summarized kinetic data of the triple DHPLC measurements of the formation of the hemiacetal of AdPym with 2‐propanol using the unified equation of chromatography.
**Figure S2.6.7.** Eyring plot for the determination of the activation parameters ΔH, ΔS, and ΔG of the hemiacetal formation with isopropanol obtained from the DHPLC experiment with the Soai aldehyde AdPym‐CHO. The upper and lower curves represent the error bands of the linear regression with a level of confidence of 95%.
**Figure S2.7.1.** DHPLC profile obtained from temperature‐dependent measurements of the pyridine‐based aldehyde Pym‐CHO in a n‐hexane/iPrOH (60/40) mobile phase at 10°C.
**Figure S2.7.2.** DHPLC profile obtained from temperature‐dependent measurements of the pyridine‐based aldehyde Pym‐CHO in a n‐hexane/iPrOH (60/40) mobile phase at 20°C.
**Figure S2.7.3.** DHPLC profile obtained from temperature‐dependent measurements of the pyridine‐based aldehyde Pym‐CHO in a n‐hexane/iPrOH (60/40) mobile phase at 30°C.
**Figure S2.7.4.** DHPLC profile obtained from temperature‐dependent measurements of the pyridine‐based aldehyde Pym‐CHO in a n‐hexane/iPrOH (60/40) mobile phase at 40°C.
**Figure S2.7.5.** DHPLC profile obtained from temperature‐dependent measurements of the pyridine‐based aldehyde Pym‐CHO in a n‐hexane/iPrOH (60/40) mobile phase at 50°C.
**Figure S2.7.6.** DHPLC profile obtained from temperature‐dependent measurements of the pyridine‐based aldehyde Pym‐CHO in a n‐hexane/iPrOH (60/40) mobile phase at 60°C.
**Figure S2.7.7.** DHPLC profile obtained from temperature‐dependent measurements of the pyridine‐based aldehyde Pym‐CHO in a n‐hexane/iPrOH (60/40) mobile phase at 60°C.
**Table S2.7.1.** Summarized experimental chromatographic parameters of the triple DHPLC measurements of the formation of the hemiacetal of Pym with 2‐propanol.
**Table S2.7.2.** Summarized kinetic data of the triple DHPLC measurements of the formation of the hemiacetal of Pym with 2‐propanol using the unified equation of chromatography.
**Figure S2.6.7.** Eyring plot for the determination of the activation parameters ΔH, ΔS, and ΔG of the hemiacetal formation with isopropanol obtained from the DHPLC experiment with the Soai aldehyde Pym‐CHO. The upper and lower curves represent the error bands of the linear regression with a level of confidence of 95%.
**Figure S3.1.** ^1^H‐NMR (CDCl3, 400 MHz) of 6‐((trimethylsilyl)ethynyl)nicotinaldehyde (TMSPyr‐CHO).
**Figure S3.2.** 13C‐NMR (CDCl3, 400 MHz) of 6‐((trimethylsilyl)ethynyl)nicotinaldehyde (TMSPyr‐CHO).
**Figure S3.3.** ^1^H‐NMR (CDCl3, 400 MHz) of 5‐bromo‐2‐((trimethylsilyl)ethynyl) pyrimidine (TMSPym‐Br).
**Figure S3.4.** 13C‐NMR (CDCl3, 400 MHz) of 5‐bromo‐2‐((trimethylsilyl)ethynyl) pyrimidine (TMSPym‐Br).
**Figure S3.5.** ^1^H‐NMR (CDCl3, 400 MHz) of 2‐(ethynyltrimethyllsilane)‐pyrimidine‐5‐carbaldehyde (TMSPym‐CHO).
**Figure S3.6.** 13C‐NMR (CDCl3, 400 MHz) of 2‐(ethynyltrimethyllsilane)‐pyrimidine‐5‐carbaldehyde (TMSPym‐CHO).
**Figure S3.7.** ^1^H‐NMR (CDCl3, 400 MHz) of ethinyl adamantane.
**Figure S3.8.** 13C NMR (CDCl3, 400 MHz) of ethinyl adamantane.
**Figure S3.9.** ^1^H‐NMR (CDCl3, 400 MHz) of 6‐((adamantan‐1‐yl)ethynyl)nicotinaldehyde (AdPyr‐CHO).
**Figure S3.10.** 13C‐NMR (CDCl3, 400 MHz) of 6‐((adamantan‐1‐yl)ethynyl)nicotinaldehyde (AdPyr‐CHO).
**Figure S3.11.** ^1^H‐NMR (CDCl3, 400 MHz) of 2‐(ethynyl‐adamantyl)‐pyrimidine‐5‐carbaldehyde (AdPym‐CHO).
**Figure S3.12.** 13C‐NMR (CDCl3, 400 MHz) of 2‐(ethynyl‐adamantyl)‐pyrimidine‐5‐carbaldehyde (AdPym‐CHO).

## Data Availability

The authors have nothing to report.
