# Supplementary material for: Stereodynamics of Hemiacetal Formation in Pyridine‐3‐Carbaldehyde and Pyrimidine‐5‐Carbaldehyde Derivatives: Implications for the Soai Reaction
Source: Chirality. 2026 Oct 3;38(10):e70152. doi: 10.1002/chir.70152 (PMC13633548; doi:10.1002/chir.70152)
Supplement: Supplementary file 1 [file CHIR-38-e70152-s001.docx]

**Supporting Information**

**Kinetic Investigations on the dynamic Hemiacetal Formation of Soai Aldehydes**

Patrick Möhler,^1^ Alexander F. Siegle^1^ and Oliver Trapp^1,^*

**Affiliations:**

^1^Department of Chemistry, Ludwig-Maximilians-University Munich, Munich, Germany.

*Corresponding author: Email: [oliver.trapp@cup.uni-muenchen.de](mailto:oliver.trapp@cup.uni-muenchen.de)

Content

[1	NMR Hemiacetal formation studies	3](#_Toc240474869)

[1.1	Hemiacetal formation in MeOD-*d_4_*	3](#_Toc240474870)

[1.2	Hemiacetal formation in *i*PrOD-*d_8_*	5](#_Toc240474871)

[2	Dynamic HPLC measurements of hemiacetal formation	7](#_Toc240474872)

[2.1	Enantioselective dynamic HPLC measurements	7](#_Toc240474873)

[2.2	Evaluation of the dynamic HPLC profiles	8](#_Toc240474874)

[2.3	DHPLC measurements of Soai aldehyde TMSPyr-CHO	9](#_Toc240474875)

[2.4	DHPLC measurements of *Soai* aldehyde TMSPym-CHO	15](#_Toc240474876)

[2.5	DHPLC measurements of *Soai* aldehyde AdPyr-CHO	21](#_Toc240474877)

[2.6	DHPLC measurements of *Soai* aldehyde AdPym-CHO	28](#_Toc240474878)

[2.7	DHPLC measurements of *Soai* aldehyde Pym-CHO	34](#_Toc240474879)

[3	NMR spectra	41](#_Toc240474880)

[4	References	47](#_Toc240474881)

# 1 NMR Hemiacetal formation studies

## 1.1 Hemiacetal formation in MeOD-*d_4_*

**Figure 1.1.1:** 1 H NMR (400 MHz) of aldehyde **TMSPyr** in MeOD-*d_4_* after 60h.

**Figure 1.1.2:** 1 H NMR (400 MHz) of aldehyde **AdPyr** in MeOD-*d_4_* after 60h.

**Figure 1.1.3:** 1 H NMR (400 MHz) of aldehyde **AdPym** in MeOD-*d_4_* after 60h.

**Figure 1.1.4:** 1 H NMR (400 MHz) of aldehyde **TMSPym** in MeOD-*d_4_* after 60h.

## 1.2 Hemiacetal formation in *i*PrOD-*d_8_*

**Figure 1.2.1:** 1 H NMR (400 MHz) of aldehyde **TMSPyr** in *i*PrOD-*d_8_* after 60h.

**Figure 1.2.2**: 1 H NMR (400 MHz) of aldehyde **AdPyr** in *i*PrOD-*d_8_* after 60h.

**Figure 1.2.3:** 1 H NMR (400 MHz) of aldehyde **TMSPym** in *i*PrOD-*d_8_* after 60h.

**Figure 1.2.4:** 1 H NMR (400 MHz) of aldehyde **AdPym** in *i*PrOD-*d_8_* after 60h.

# 2 Dynamic HPLC measurements of hemiacetal formation

## 2.1 Enantioselective dynamic HPLC measurements

Dynamic HPLC (DHPLC) measurements were performed on an *Agilent* 1200 Infinity HPLC equipped with a binary pump, an autosampler (*Agilent* HiP+), a thermostated column oven and a photodiode array detector (DAD). All operations were controlled by the *Agilent Chemstation* software. The separations were performed on an immobilized stationary phase using *n*-hexane/*i*PrOH = 60/40 as mobile phase at a flow rate of 1.0 mL/min. The investigated systems were conducted with the similar mobile phase to exclude solvent dependent influences on activation parameters.^[1]^ The columns were preheated for at least 30 min prior to the measurements at a specific temperature. It should be noted that the temperature-dependent measurements were conducted as the hemiacetal-formation was equilibrated at room temperature.

The on-column equilibrium between the aldehyde and the hemiacetal of the pyridine system **TMSPyr**, was monitored on a Chiralpak® IC column (250 mm, i.D. 4.6 mm, particle size: 5 μm) from *Chiral Technologies*. The equilibrium was monitored between 10 °C and 70 °C.

The on-column equilibrium between the aldehyde and the hemiacetal of the pyridine system **AdPyr**, was monitored on a Chiralpak® IA column (250 mm, i.D. 4.6 mm, particle size: 5 μm) from *Chiral Technologies*. The equilibrium was monitored between 10 °C and 70 °C.

The on-column equilibrium between the aldehyde and the hemiacetal of the pyridine system **TMSPym**, was monitored on a Chiralpak® IB column (250 mm, i.D. 4.6 mm, particle size: 5 μm) from *Chiral Technologies*. The equilibrium was monitored between 10 °C and 60 °C.

The on-column equilibrium between the aldehyde and the hemiacetal of the pyridine system **AdPym**, was monitored on a Chiralpak® IB column (250 mm, i.D. 4.6 mm, particle size: 5 μm) from *Chiral Technologies*. The equilibrium was monitored between 10 °C and 60 °C.

The on column equilibrium between aldehyde and hemiacetal of the unsubstituted pyirimidine-5-carbaldehyde **Pym** was monitored on a Chiralpak® IC column (250 mm, i.D. 4.6 mm, particle size: 5 μm) from *Chiral Technologies*. The equilibrium was monitored between 10 °C and 70 °C.

## 2.2 Evaluation of the dynamic HPLC profiles

The evaluation of chromatographic profiles obtained from the DHPLC measurements was conducted according to a previously described method.^[2]^ The rate constants *k* of hemiacetal formation were determined by analysis of the chromatographic peak profiles using the unified equation of dynamic chromatography implemented in the software *DCXplorer*.^[3, 4]^ The unified equation allows the direct calculation of reaction rate constants and Gibbs activation energies ∆G^╪^ for all types of first-order reactions taking place in chromatographic systems. A detailed description is given in the literature.^[3]^

## 2.3 **DHPLC measurements of Soai aldehyde TMSPyr-CHO**

**Figure 2.3.1**: DHPLC profile obtained from temperature dependent measurements of the pyridine-based aldehyde **TMSPyr-CHO** in a n-hexane/*i*PrOH (60/40) mobile Phase at 20°C.

**Figure 2.3.2**: DHPLC profile obtained from temperature dependent measurements of the pyridine-based aldehyde **TMSPyr-CHO** in a n-hexane/iPrOH (60/40) mobile Phase at 30°C.

**Figure 2.3.3**: DHPLC profile obtained from temperature dependent measurements of the pyridine-based aldehyde **TMSPyr-CHO** in a n-hexane/iPrOH (60/40) mobile Phase at 40°C.

**Figure 2.3.4**: DHPLC profile obtained from temperature dependent measurements of the pyridine-based aldehyde **TMSPyr-CHO** in a n-hexane/iPrOH (60/40) mobile Phase at 50°C.

**Figure 2.3.5**: DHPLC profile obtained from temperature dependent measurements of the pyridine-based aldehyde **TMSPyr-CHO** in a n-hexane/iPrOH (60/40) mobile Phase at 60°C

**Figure 2.3.6**: DHPLC profile obtained from temperature dependent measurements of the pyridine-based aldehyde **TMSPyr-CHO** in a n-hexane/iPrOH (60/40) mobile Phase at 70°C.

**Figure: 2.3.7**: DHPLC profile obtained from temperature dependent measurements of the pyridine-based aldehyde **TMSPyr-CHO** in a n-hexane/iPrOH (60/40) mobile Phase at 80°C.

**Table 2.3.1:** Summarized experimental chromatographic parameters of the triple DHPLC measurements of the formation of the hemiacetal of **TMSPyr** with 2-propanol.

| **Temperature [°C]** | **Retention time** | | **Half width** | | **Height** | | | **Area** | |
| --- | --- | --- | --- | --- | --- | --- | --- | --- | --- |
|  | **t1 [min]** | **t2 [min]** | **w1 [s]** | **w2 [s]** | **Peak 1** | **Plateau** | **Peak 2** | **Peak 1** | **Peak 2** |
| 20 | 2,439 | 5,632 | 5,2 | 10,8 | 96,35 | 0,13 | 100,00 | 37,66 | 62,34 |
| 20 | 2,444 | 5,644 | 5,2 | 10,8 | 96,31 | 0,15 | 100,00 | 37,52 | 62,48 |
| 20 | 2,445 | 5,652 | 5,2 | 10,8 | 96,56 | 0,13 | 100,00 | 37,67 | 62,33 |
| 30 | 2,365 | 4,769 | 5,6 | 8,0 | 69,43 | 0,27 | 100,00 | 36,67 | 63,33 |
| 30 | 2,372 | 4,772 | 5,6 | 8,0 | 69,26 | 0,28 | 100,00 | 36,51 | 63,49 |
| 30 | 2,369 | 4,769 | 5,6 | 8,4 | 69,02 | 0,33 | 100,00 | 36,52 | 63,48 |
| 40 | 2,309 | 4,155 | 6,4 | 6,8 | 50,12 | 0,45 | 100,00 | 34,57 | 65,43 |
| 40 | 2,310 | 4,154 | 6,4 | 6,4 | 49,61 | 0,43 | 100,00 | 34,4 | 65,6 |
| 40 | 2,310 | 4,150 | 6,4 | 6,4 | 49,36 | 0,47 | 100,00 | 34,37 | 65,63 |
| 50 | 2,257 | 3,697 | 7,2 | 5,6 | 34,51 | 0,95 | 100,00 | 30,19 | 69,81 |
| 50 | 2,260 | 3,697 | 7,2 | 5,6 | 34,02 | 1,02 | 100,00 | 29,99 | 70,01 |
| 50 | 2,259 | 3,692 | 6,8 | 5,6 | 33,71 | 1,06 | 100,00 | 29,83 | 70,17 |
| 60 | 2,224 | 3,397 | 7,6 | 5,2 | 21,81 | 1,46 | 100,00 | 24,13 | 75,87 |
| 60 | 2,224 | 3,397 | 7,2 | 5,2 | 20,86 | 1,37 | 100,00 | 22,65 | 77,35 |
| 60 | 2,225 | 3,395 | 7,6 | 5,2 | 20,62 | 1,37 | 100,00 | 22,61 | 77,39 |
| 70 | 2,177 | 3,124 | 7,2 | 4,8 | 9,62 | 2,37 | 100,00 | 15,6 | 84,4 |
| 70 | 2,182 | 3,129 | 7,2 | 4,8 | 9,34 | 2,33 | 100,00 | 15,35 | 84,65 |
| 70 | 2,182 | 3,129 | 7,2 | 4,8 | 8,99 | 2,24 | 100,00 | 14,73 | 85,27 |
| 80 | 2,144 | 2,927 | 6,8 | 4,4 | 2,97 | 2,73 | 100,00 | 8,66 | 91,34 |
| 80 | 2,147 | 2,930 | 6,8 | 4,4 | 2,88 | 2,74 | 100,00 | 8,59 | 91,41 |
| 80 | 2,147 | 2,930 | 6,8 | 4,8 | 2,89 | 2,78 | 100,00 | 8,62 | 91,38 |

**Table 2.3.2:** Summarized kinetic data of the triple DHPLC measurements of the formation of the hemiacetal of **TMSPyr** with 2-propanol using the unified equation of chromatography

| **Forward Reaction** | | **Backward Reaction** | |
| --- | --- | --- | --- |
| **1/T [1/K]** | **ln(k/T)** | **1/T [1/K]** | **ln(k/T)** |
| 0,003411 | -14,992 | 0,003411 | -15,496 |
| 0,003411 | -15,14 | 0,003411 | -15,65 |
| 0,003411 | -14,9471 | 0,003411 | -15,4506 |
| 0,003299 | -14,0264 | 0,003299 | -14,5728 |
| 0,003299 | -14,0783 | 0,003299 | -14,6316 |
| 0,003299 | -13,9991 | 0,003299 | -14,552 |
| 0,003193 | -13,5224 | 0,003193 | -14,1604 |
| 0,003193 | -13,5371 | 0,003193 | -14,1826 |
| 0,003193 | -13,4556 | 0,003193 | -14,1025 |
| 0,003095 | -12,9672 | 0,003095 | -13,8054 |
| 0,003095 | -13,0024 | 0,003095 | -13,8501 |
| 0,003095 | -12,9245 | 0,003095 | -13,7799 |
| 0,003002 | -12,5407 | 0,003002 | -13,6863 |
| 0,003002 | -12,6174 | 0,003002 | -13,8456 |
| 0,003002 | -12,5004 | 0,003002 | -13,7309 |
| 0,002914 | -11,3832 | 0,002914 | -13,0714 |
| 0,002914 | -11,3233 | 0,002914 | -13,0307 |
| 0,002914 | -11,3862 | 0,002914 | -13,1422 |
| 0,002832 | -10,5599 | 0,002832 | -12,9158 |
| 0,002832 | -10,5444 | 0,002832 | -12,9092 |
| 0,002832 | -10,5677 | 0,002832 | -12,9286 |

**Figurfe 2.3.8**: Eyring plot for the determination of the activation parameters ΔH, ΔS and ΔG of the hemiacetal formation with isopropanol obtained from the DHPLC experiment with the *Soai* aldehyde **TMSPyr-CHO**. The upper and lower curves represent the error bands of the linear regression with a level of confidence of 95%.

## 2.4 DHPLC measurements of *Soai* aldehyde TMSPym-CHO

**Figure 2.4.1**: DHPLC profile obtained from temperature dependent measurements of the pyridine-based aldehyde **TMSPym-CHO** in a n-hexane/iPrOH (60/40) mobile Phase at 20°C.

**Figure 2.4.2**: DHPLC profile obtained from temperature dependent measurements of the pyridine-based aldehyde **TMSPym-CHO** in a n-hexane/iPrOH (60/40) mobile Phase at 30°C

**Figure 2.4.3**: DHPLC profile obtained from temperature dependent measurements of the pyridine-based aldehyde **TMSPym-CHO** in a n-hexane/iPrOH (60/40) mobile Phase at 40°C.

**Figure 2.4.4:** DHPLC profile obtained from temperature dependent measurements of the pyridine-based aldehyde **TMSPym-CHO** in a n-hexane/iPrOH (60/40) mobile Phase at 50°C.

**Figure 2.4.5**: DHPLC profile obtained from temperature dependent measurements of the pyridine-based aldehyde **TMSPym-CHO** in a n-hexane/iPrOH (60/40) mobile Phase at 60°C.

**Figure 2.4.6**: DHPLC profile obtained from temperature dependent measurements of the pyridine-based aldehyde **TMSPym-CHO** in a n-hexane/iPrOH (60/40) mobile Phase at 70°C.

**Table 2.4.1:** Summarized experimental chromatographic parameters of the triple DHPLC measurements of the formation of the hemiacetal of **TMSPym** with 2-propanol.

| **Temperature**  **[°C]** | **Retentiom time** | | **Half width** | | **Height** | | | **Area** | |
| --- | --- | --- | --- | --- | --- | --- | --- | --- | --- |
|  | **t1 [min]** | **t2 [min]** | **w1 [s]** | **w2 [s]** | **Peak 1** | **Plateau** | **Peak 2** | **Peak 1** | **Peak 2** |
| 20 | 2,193 | 3,999 | 2,8 | 7,6 | 100,00 | 1,09 | 2,48 | 87,86 | 12,14 |
| 20 | 2,196 | 4,003 | 3,2 | 7,6 | 100,00 | 1,13 | 2,43 | 88,16 | 11,84 |
| 20 | 2,193 | 4,003 | 2,8 | 7,2 | 100,00 | 1,01 | 2,30 | 89,24 | 10,76 |
| 30 | 2,156 | 3,596 | 2,8 | 6,8 | 100,00 | 2,16 | 3,18 | 86,25 | 13,75 |
| 30 | 2,158 | 3,594 | 3,2 | 7,2 | 100,00 | 2,17 | 3,21 | 86,14 | 13,86 |
| 30 | 2,158 | 3,594 | 3,2 | 6,8 | 100,00 | 2,23 | 3,25 | 85,81 | 14,19 |
| 40 | 2,124 | 3,298 | 2,8 | 6,4 | 100,00 | 4,95 | 5,51 | 81,14 | 18,86 |
| 40 | 2,124 | 3,294 | 2,8 | 6,4 | 100,00 | 5,10 | 5,55 | 81,07 | 18,93 |
| 40 | 2,126 | 3,296 | 3,2 | 6,0 | 100,00 | 4,91 | 5,41 | 81,75 | 18,25 |
| 50 | 2,101 | 3,064 | 3,6 | 5,6 | 100,00 | 11,56 | 9,91 | 75,5 | 24,5 |
| 50 | 2,101 | 3,061 | 3,6 | 5,6 | 100,00 | 11,74 | 10,03 | 75,23 | 24,77 |
| 50 | 2,101 | 3,061 | 3,6 | 5,6 | 100,00 | 11,89 | 10,22 | 74,97 | 25,03 |
| 60 | 2,081 | 2,881 | 3,6 | 5,2 | 100,00 | 29,00 | 19,36 | 67,55 | 32,45 |
| 60 | 2,079 | 2,879 | 3,2 | 5,2 | 100,00 | 29,57 | 19,74 | 67,02 | 32,98 |
| 60 | 2,078 | 2,878 | 3,2 | 5,6 | 100,00 | 29,52 | 19,64 | 67,06 | 32,94 |
| 70 | 2,063 | 2,743 | 3,6 | 5,6 | 100,00 | 68,78 | 39,47 | 59,57 | 40,43 |
| 70 | 2,063 | 2,743 | 3,6 | 5,6 | 100,00 | 69,80 | 40,58 | 59,01 | 40,99 |
| 70 | 2,061 | 2,744 | 3,6 | 5,6 | 100,00 | 70,79 | 42,58 | 58,13 | 41,87 |

**Table 2.4.2:** Summarized kinetic data of the triple DHPLC measurements of the formation of the hemiacetal of **TMSPym** with 2-propanol using the unified equation of chromatography.

| **Forward Reaction** | | **Backward Reaction** | |
| --- | --- | --- | --- |
| **1/T [1/K]** | **ln(k/T)** | **1/T [1/K]** | **ln(k/T)** |
| 0,003411 | -11,7026 | 0,003411 | -9,72337 |
| 0,003411 | -11,7945 | 0,003411 | -9,78687 |
| 0,003411 | -11,7555 | 0,003411 | -9,64004 |
| 0,003299 | -11,2332 | 0,003299 | -9,39698 |
| 0,003299 | -11,4648 | 0,003299 | -9,63782 |
| 0,003299 | -11,4452 | 0,003299 | -9,64558 |
| 0,003193 | -10,918 | 0,003193 | -9,45888 |
| 0,003193 | -10,9028 | 0,003193 | -9,44824 |
| 0,003193 | -11,0266 | 0,003193 | -9,52707 |
| 0,003095 | -10,6875 | 0,003095 | -9,56205 |
| 0,003095 | -10,6794 | 0,003095 | -9,56849 |
| 0,003095 | -10,6724 | 0,003095 | -9,57542 |
| 0,003002 | -10,2636 | 0,003002 | -9,53045 |
| 0,003002 | -10,1962 | 0,003002 | -9,48715 |
| 0,003002 | -10,197 | 0,003002 | -9,48614 |
| 0,002914 | -9,86954 | 0,002914 | -9,48196 |
| 0,002914 | -9,8656 | 0,002914 | -9,50122 |
| 0,002914 | -9,86672 | 0,002914 | -9,53861 |

**Fig. 2.4.7: E**yring plot for the determination of the activation parameters ΔH, ΔS and ΔG of the hemiacetal formation with isopropanol obtained from the DHPLC experiment with the *Soai* aldehyde **TMSPym-CHO**. The upper and lower curves represent the error bands of the linear regression with a level of confidence of 95%.

## 2.5 DHPLC measurements of *Soai* aldehyde AdPyr-CHO

**Figure 2.5.1:** DHPLC profile obtained from temperature dependent measurements of the pyridine-based aldehyde **AdPyr-CHO** in a n-hexane/iPrOH (60/40) mobile Phase at 10°C.

**Figure 2.5.2:** DHPLC profile obtained from temperature dependent measurements of the pyridine-based aldehyde **AdPyr-CHO** in a n-hexane/iPrOH (60/40) mobile Phase at 20°C.

**Figure 2.5.3:** DHPLC profile obtained from temperature dependent measurements of the pyridine-based aldehyde **AdPyr-CHO** in a n-hexane/iPrOH (60/40) mobile Phase at 30°C.

**Figure 2.5.4:** DHPLC profile obtained from temperature dependent measurements of the pyridine-based aldehyde **AdPyr-CHO** in a n-hexane/iPrOH (60/40) mobile Phase at 40°C.

**Figure 2.5.5:** DHPLC profile obtained from temperature dependent measurements of the pyridine-based aldehyde **AdPyr-CHO** in a n-hexane/iPrOH (60/40) mobile Phase at 50°C.

**Figure 2.5.6:** DHPLC profile obtained from temperature dependent measurements of the pyridine-based aldehyde **AdPyr-CHO** in a n-hexane/iPrOH (60/40) mobile Phase at 60°C.

**Figure 2.5.7:** DHPLC profile obtained from temperature dependent measurements of the pyridine-based aldehyde **AdPyr-CHO** in a n-hexane/iPrOH (60/40) mobile phase at 70°C.

**Table 2.5.1:** Summarized experimental chromatographic parameters of the triple DHPLC measurements of the formation of the hemiacetal of **AdPyr** with 2-propanol.

| **Temperature**  **[°C]** | **Retentiom time** | | **Half width** | | **Height** | | | **Area** | |
| --- | --- | --- | --- | --- | --- | --- | --- | --- | --- |
|  | **t1 [min]** | **t2 [min]** | **w1 [s]** | **w2 [s]** | **Peak 1** | **Plateau** | **Peak 2** | **Peak 1** | **Peak 2** |
| 10 | 3,919 | 7,859 | 7,6 | 12,8 | 66,24 | 0,04 | 100,00 | 30,02 | 69,98 |
| 10 | 3,923 | 7,859 | 7,6 | 12,8 | 66,26 | 0,04 | 100,00 | 29,91 | 70,09 |
| 10 | 3,919 | 7,859 | 7,6 | 12,8 | 66,32 | 0,05 | 100,00 | 29,86 | 70,14 |
| 20 | 3,826 | 6,929 | 6,8 | 10,0 | 57,88 | 0,06 | 100,00 | 29,92 | 70,08 |
| 20 | 3,824 | 6,921 | 6,8 | 10,4 | 57,87 | 0,06 | 100,00 | 29,93 | 70,07 |
| 20 | 3,824 | 6,921 | 6,8 | 10,0 | 57,84 | 0,06 | 100,00 | 29,81 | 70,19 |
| 30 | 3,746 | 6,209 | 6,8 | 8,8 | 49,28 | 0,10 | 100,00 | 29,16 | 70,84 |
| 30 | 3,744 | 6,204 | 6,8 | 8,8 | 48,95 | 0,13 | 100,00 | 28,59 | 71,41 |
| 30 | 3,743 | 6,203 | 6,4 | 8,4 | 48,97 | 0,12 | 100,00 | 28,79 | 71,21 |
| 40 | 3,681 | 5,651 | 6,8 | 7,2 | 40,46 | 0,30 | 100,00 | 27,3 | 72,7 |
| 40 | 3,679 | 5,646 | 6,8 | 7,6 | 40,28 | 0,35 | 100,00 | 27,3 | 72,7 |
| 40 | 3,679 | 5,649 | 6,8 | 7,2 | 40,16 | 0,33 | 100,00 | 26,99 | 73,01 |
| 50 | 3,624 | 5,218 | 6,0 | 6,8 | 29,97 | 0,87 | 100,00 | 23,77 | 76,23 |
| 50 | 3,624 | 5,214 | 6,0 | 6,8 | 29,73 | 0,73 | 100,00 | 23,7 | 76,3 |
| 50 | 3,626 | 5,216 | 6,0 | 6,8 | 29,49 | 0,68 | 100,00 | 23,19 | 76,81 |
| 60 | 3,483 | 4,866 | 5,2 | 6,0 | 4,85 | 1,27 | 100,00 | 19,33 | 80,67 |
| 60 | 3,481 | 4,864 | 4,8 | 6,0 | 4,82 | 1,20 | 100,00 | 19,23 | 80,77 |
| 60 | 3,481 | 4,864 | 4,8 | 6,0 | 4,82 | 1,22 | 100,00 | 19,2 | 80,8 |
| 70 | 3,414 | 4,591 | 4,4 | 5,6 | 4,12 | 1,37 | 100,00 | 13,27 | 86,73 |
| 70 | 3,413 | 4,586 | 4,0 | 5,6 | 3,97 | 1,33 | 100,00 | 12,66 | 87,34 |
| 70 | 3,413 | 4,589 | 4,0 | 5,6 | 4,14 | 1,54 | 100,00 | 13,31 | 86,69 |

**Table 2.5.2:** Summarized kinetic data of the triple DHPLC measurements of the formation of the hemiacetal of **AdPyr** with 2-propanol using the unified equation of chromatography.

| **Forward Reaction** | | **Backward Reaction** | |
| --- | --- | --- | --- |
| **1/T [1/K]** | **ln(k/T)** | **1/T [1/K]** | **ln(k/T)** |
| 0,003532 | -16,3732 | 0,003532 | -17,2195 |
| 0,003532 | -16,3718 | 0,003532 | -17,2234 |
| 0,003532 | -16,1488 | 0,003532 | -17,0027 |
| 0,003411 | -16,0094 | 0,003411 | -16,8605 |
| 0,003411 | -16,0094 | 0,003411 | -16,8601 |
| 0,003411 | -15,9684 | 0,003411 | -16,8247 |
| 0,003299 | -15,568 | 0,003299 | -16,4556 |
| 0,003299 | -15,2969 | 0,003299 | -16,2123 |
| 0,003299 | -15,3337 | 0,003299 | -16,2393 |
| 0,003193 | -14,4895 | 0,003193 | -15,469 |
| 0,003193 | -14,3911 | 0,003193 | -15,3706 |
| 0,003193 | -14,3906 | 0,003193 | -15,3857 |
| 0,003095 | -13,567 | 0,003095 | -14,7324 |
| 0,003095 | -13,7403 | 0,003095 | -14,9095 |
| 0,003095 | -13,8055 | 0,003095 | -15,0031 |
| 0,003002 | -13,158 | 0,003002 | -14,5867 |
| 0,003002 | -13,2162 | 0,003002 | -14,6514 |
| 0,003002 | -13,2002 | 0,003002 | -14,6373 |
| 0,002914 | -13,1147 | 0,002914 | -14,992 |
| 0,002914 | -13,1356 | 0,002914 | -15,0669 |
| 0,002914 | -13,0051 | 0,002914 | -14,8789 |

**Figure 2.5.8:** Eyring plot for the determination of the activation parameters ΔH, ΔS and ΔG of the hemiacetal formation with isopropanol obtained from the DHPLC experiment with the *Soai* aldehyde **AdPyr-CHO**. The upper and lower curves represent the error bands of the linear regression with a level of confidence of 95%.

## 2.6 DHPLC measurements of *Soai* aldehyde AdPym-CHO

**Figure 2.6.1:** DHPLC profile obtained from temperature dependent measurements of the pyridine-based aldehyde **AdPym-CHO** in a n-hexane/iPrOH (60/40) mobile phase at 10°C.

**Figure 2.6.2:** DHPLC profile obtained from temperature dependent measurements of the pyridine-based aldehyde **AdPym-CHO** in a n-hexane/iPrOH (60/40) mobile phase at 20°C.

**Figure 2.6.3:** DHPLC profile obtained from temperature dependent measurements of the pyridine-based aldehyde AdPym-CHO in a n-hexane/iPrOH (60/40) mobile phase at 30°C.

**Figure 2.6.4:** DHPLC profile obtained from temperature dependent measurements of the pyridine-based aldehyde AdPym-CHO in a n-hexane/iPrOH (60/40) mobile phase at 40°C.

**Figure 2.6.5:** DHPLC profile obtained from temperature dependent measurements of the pyridine-based aldehyde AdPym-CHO in a n-hexane/iPrOH (60/40) mobile phase at 50°C.

**Figure 2.6.6:** DHPLC profile obtained from temperature dependent measurements of the pyridine-based aldehyde AdPym-CHO in a n-hexane/iPrOH (60/40) mobile phase at 60°C.

**Table 2.6.1:** Summarized experimental chromatographic parameters of the triple DHPLC measurements of the formation of the hemiacetal of **AdPym** with 2-propanol.

| **Temperature [°C]** | **Retention time** | | **Half width** | | **Height** | | | **Area** | |
| --- | --- | --- | --- | --- | --- | --- | --- | --- | --- |
|  | **t1 [min]** | **t2 [min]** | **w1 [s]** | **w2 [s]** | **Peak 1** | **Plateau** | **Peak 2** | **Peak 1** | **Peak 2** |
| 10 | 4,023 | 9,263 | 5,0 | 15,2 | 100,00 | 1,19 | 19,07 | 74,33 | 25,67 |
| 10 | 4,026 | 9,263 | 5,5 | 15,2 | 100,00 | 1,18 | 18,98 | 74,67 | 25,33 |
| 10 | 4,028 | 9,261 | 5,5 | 15,2 | 100,00 | 1,24 | 19,05 | 74,28 | 25,72 |
| 20 | 3,918 | 8,264 | 4,6 | 12,2 | 100,00 | 2,20 | 21,64 | 72,91 | 27,09 |
| 20 | 3,918 | 8,248 | 5,0 | 12,2 | 100,00 | 2,10 | 21,73 | 72,94 | 27,06 |
| 20 | 3,918 | 8,251 | 5,0 | 12,2 | 100,00 | 2,31 | 21,73 | 72,79 | 27,21 |
| 30 | 3,833 | 7,493 | 4,6 | 10,6 | 100,00 | 4,40 | 26,13 | 69,52 | 30,48 |
| 30 | 3,829 | 7,483 | 4,6 | 10,6 | 100,00 | 4,43 | 26,37 | 69,15 | 30,85 |
| 30 | 3,838 | 7,528 | 5,0 | 10,6 | 100,00 | 4,06 | 26,11 | 69,39 | 30,61 |
| 40 | 3,711 | 6,528 | 4,2 | 8,7 | 100,00 | 12,26 | 48,98 | 60,55 | 39,45 |
| 40 | 3,708 | 6,508 | 4,2 | 8,7 | 100,00 | 13,18 | 50,33 | 60,41 | 39,59 |
| 40 | 3,708 | 6,501 | 4,6 | 8,7 | 100,00 | 13,41 | 51,54 | 59,68 | 40,32 |
| 50 | 3,636 | 5,963 | 4,6 | 8,4 | 92,94 | 39,56 | 100,00 | 50,92 | 49,08 |
| 50 | 3,634 | 5,954 | 4,2 | 8,0 | 89,23 | 39,60 | 100,00 | 50,31 | 49,69 |
| 50 | 3,634 | 5,954 | 4,2 | 8,4 | 89,23 | 40,82 | 100,00 | 50,54 | 49,46 |
| 60 | 3,581 | 5,534 | 4,2 | 8,0 | 26,44 | 51,48 | 100,00 | 39,41 | 60,59 |
| 60 | 3,583 | 5,526 | 5,0 | 8,0 | 27,54 | 53,75 | 100,00 | 39,84 | 60,16 |
| 60 | 3,581 | 5,528 | 4,6 | 8,0 | 26,40 | 52,92 | 100,00 | 39,46 | 60,54 |

**Table 2.6.2:** Summarized kinetic data of the triple DHPLC measurements of the formation of the hemiacetal of **AdPym** with 2-propanol using the unified equation of chromatography.

| **Forward Reaction** | | **Backward Reaction** | |
| --- | --- | --- | --- |
| **1/T [1/K]** | **ln(k/T)** | **1/T [1/K]** | **ln(k/T)** |
| 0,003411 | -11,9749 | 0,003411 | -10,9117 |
| 0,003411 | -12,0608 | 0,003411 | -10,9797 |
| 0,003411 | -12,0226 | 0,003411 | -10,962 |
| 0,003299 | -11,3712 | 0,003299 | -10,3812 |
| 0,003299 | -11,6985 | 0,003299 | -10,7069 |
| 0,003299 | -11,6273 | 0,003299 | -10,6433 |
| 0,003193 | -11,2684 | 0,003193 | -10,4438 |
| 0,003193 | -11,2694 | 0,003193 | -10,4623 |
| 0,003193 | -11,3777 | 0,003193 | -10,5593 |
| 0,003095 | -10,8176 | 0,003095 | -10,3892 |
| 0,003095 | -10,7811 | 0,003095 | -10,3585 |
| 0,003095 | -10,828 | 0,003095 | -10,4359 |
| 0,003002 | -10,7214 | 0,003002 | -10,6846 |
| 0,003002 | -10,6874 | 0,003002 | -10,675 |
| 0,003002 | -10,694 | 0,003002 | -10,6724 |
| 0,002914 | -10,5013 | 0,002914 | -10,9314 |
| 0,002914 | -10,4795 | 0,002914 | -10,8916 |
| 0,002914 | -10,4846 | 0,002914 | -10,9126 |

**Figure 2.6.7:** Eyring plot for the determination of the activation parameters ΔH, ΔS and ΔG of the hemiacetal formation with isopropanol obtained from the DHPLC experiment with the *Soai* aldehyde **AdPym-CHO**. The upper and lower curves represent the error bands of the linear regression with a level of confidence of 95%.

## 2.7 DHPLC measurements of *Soai* aldehyde Pym-CHO

**Figure 2.7.1:** DHPLC profile obtained from temperature dependent measurements of the pyridine-based aldehyde **Pym-CHO** in a n-hexane/iPrOH (60/40) mobile phase at 10°C.

**Figure 2.7.2:** DHPLC profile obtained from temperature dependent measurements of the pyridine-based aldehyde **Pym-CHO** in a n-hexane/iPrOH (60/40) mobile phase at 20°C.

**Figure 2.7.3:** DHPLC profile obtained from temperature dependent measurements of the pyridine-based aldehyde **Pym-CHO** in a n-hexane/iPrOH (60/40) mobile phase at 30°C.

**Figure 2.7.4:** DHPLC profile obtained from temperature dependent measurements of the pyridine-based aldehyde **Pym-CHO** in a n-hexane/iPrOH (60/40) mobile phase at 40°C.

**Figure 2.7.5:** DHPLC profile obtained from temperature dependent measurements of the pyridine-based aldehyde **Pym-CHO** in a n-hexane/iPrOH (60/40) mobile phase at 50°C.

**Figure 2.7.6:** DHPLC profile obtained from temperature dependent measurements of the pyridine-based aldehyde **Pym-CHO** in a n-hexane/iPrOH (60/40) mobile phase at 60°C.

**Figure 2.7.7:** DHPLC profile obtained from temperature dependent measurements of the pyridine-based aldehyde **Pym-CHO** in a n-hexane/iPrOH (60/40) mobile phase at 60°C.

**Table 2.7.1:** Summarized experimental chromatographic parameters of the triple DHPLC measurements of the formation of the hemiacetal of **Pym** with 2-propanol.

| Temperature  [°C] | Retention time | | Half width | | Height | | | Area | |
| --- | --- | --- | --- | --- | --- | --- | --- | --- | --- |
|  | **t1 [min]** | **t2 [min]** | **w1 [s]** | **w2 [s]** | **Peak 1** | **Plateau** | **Peak 2** | **Peak 1** | **Peak 2** |
| 10 | 3.234 | 8.235 | 7.2 | 16.8 | 200.00 | 1.07 | 64.46 | 119.42 | 40.29 |
| 10 | 3.234 | 8.304 | 6.8 | 16.0 | 200.00 | 1.10 | 63.03 | 119.8 | 40.1 |
| 10 | 3.234 | 8.324 | 6.8 | 16.0 | 200.00 | 1.05 | 61.44 | 121.9 | 39.05 |
| 20 | 3.027 | 6.884 | 6.0 | 12.0 | 200.00 | 1.97 | 65.57 | 123.2 | 38.4 |
| 20 | 3.001 | 6.760 | 6.0 | 11.2 | 200.00 | 2.16 | 64.17 | 123.88 | 38.06 |
| 20 | 2.992 | 6.725 | 6.0 | 12.0 | 200.00 | 2.16 | 62.65 | 125 | 37.5 |
| 30 | 2.840 | 5.776 | 4.8 | 8.8 | 200.00 | 4.11 | 66.93 | 123.4 | 38.3 |
| 30 | 2.822 | 5.669 | 4.8 | 8.8 | 200.00 | 4.58 | 66.15 | 122.8 | 38.6 |
| 30 | 2.818 | 5.645 | 4.8 | 9.6 | 200.00 | 4.80 | 65.06 | 123.04 | 38.48 |
| 40 | 2.692 | 4.919 | 5.2 | 7.2 | 200.00 | 10.29 | 73.69 | 115.7 | 42.15 |
| 40 | 2.684 | 4.870 | 5.2 | 7.2 | 200.00 | 11.16 | 71.95 | 116.46 | 41.77 |
| 40 | 2.690 | 4.870 | 4.8 | 7.2 | 200.00 | 11.21 | 70.30 | 117.1 | 41.45 |
| 50 | 2.582 | 4.298 | 5.6 | 7.2 | 200.00 | 22.58 | 74.44 | 106.56 | 46.72 |
| 50 | 2.580 | 4.263 | 5.6 | 7.2 | 200.00 | 23.36 | 73.46 | 105.6 | 47.2 |
| 50 | 2.581 | 4.277 | 5.6 | 7.2 | 200.00 | 24.53 | 72.53 | 105.4 | 47.3 |
| 60 | 2.530 | 3.883 | 8.0 | 8.0 | 84.57 | 48.63 | 100.00 | 46.39 | 53.61 |
| 60 | 2.516 | 3.830 | 8.0 | 8.0 | 75.77 | 47.51 | 100.00 | 44.01 | 55.99 |
| 60 | 2.511 | 3.818 | 7.2 | 8.0 | 75.04 | 48.75 | 100.00 | 44.4 | 55.6 |
| 70 | 2.477 | 3.490 | 8.8 | 8.8 | 45.62 | 66.05 | 100.00 | 39.95 | 60.05 |

**Table 2.7.2:** Summarized kinetic data of the triple DHPLC measurements of the formation of the hemiacetal of **Pym** with 2-propanol using the unified equation of chromatography.

| **Forward Reaction** | | **Backward Reaction** | |
| --- | --- | --- | --- |
| **1/T [1/K]** | **ln(k/T)** | **1/T [1/K]** | **ln(k/T)** |
| 3.53E-03 | -13.5255 | 3.53E-03 | -12.4390 |
| 3.53E-03 | -13.4390 | 3.53E-03 | -12.3446 |
| 3.53E-03 | -13.4710 | 3.53E-03 | -12.3327 |
| 3.41E-03 | -13.0014 | 3.41E-03 | -11.8356 |
| 3.41E-03 | -12.9273 | 3.41E-03 | -11.7471 |
| 3.41E-03 | -12.9284 | 3.41E-03 | -11.7244 |
| 3.30E-03 | -12.3152 | 3.30E-03 | -11.1453 |
| 3.30E-03 | -12.2388 | 3.30E-03 | -11.0815 |
| 3.30E-03 | -12.2020 | 3.30E-03 | -11.0396 |
| 3.19E-03 | -11.7970 | 3.19E-03 | -10.7873 |
| 3.19E-03 | -11.7342 | 3.19E-03 | -10.7088 |
| 3.19E-03 | -11.6608 | 3.19E-03 | -10.6223 |
| 3.09E-03 | -11.4144 | 3.09E-03 | -10.5899 |
| 3.09E-03 | -11.4086 | 3.09E-03 | -10.6033 |
| 3.09E-03 | -11.3546 | 3.09E-03 | -10.5533 |
| 3.00E-03 | -10.3508 | 3.00E-03 | -10.4954 |
| 3.00E-03 | -10.2619 | 3.00E-03 | -10.5027 |
| 3.00E-03 | -10.2602 | 3.00E-03 | -10.4851 |
| 2.91E-03 | -9.9671 | 2.91E-03 | -10.3747 |

**Figure 2.6.7:** Eyring plot for the determination of the activation parameters ΔH, ΔS and ΔG of the hemiacetal formation with isopropanol obtained from the DHPLC experiment with the *Soai* aldehyde **Pym-CHO**. The upper and lower curves represent the error bands of the linear regression with a level of confidence of 95%.

# 3 NMR spectra

**Figure 3.1**: ^1^H-NMR (CDCl_3_, 400 MHz) of 6-((Trimethylsilyl)ethynyl)nicotinaldehyde (**TMSPyr-CHO**).

**Figure 3.2**: ^13^C-NMR (CDCl3, 400 MHz) of 6-((Trimethylsilyl)ethynyl)nicotinaldehyde (**TMSPyr-CHO**).

**Figure 3.3**: ^1^H-NMR (CDCl_3_, 400 MHz) of 5-Bromo-2-((trimethylsilyl)ethynyl) pyrimidine (**TMSPym-Br**).

**Figure 3.4**: ^13^C-NMR (CDCl_3_, 400 MHz) of 5-Bromo-2-((trimethylsilyl)ethynyl) pyrimidine (**TMSPym-Br**).

**Figure 3.5**: ^1^H-NMR (CDCl_3_, 400 MHz) of 2-(Ethynyltrimethyllsilane)-pyrimidine-5-carbaldehyde (**TMSPym-CHO**).

**Figure 3.6:** ^13^C-NMR (CDCl_3_, 400 MHz) of 2-(Ethynyltrimethyllsilane)-pyrimidine-5-carbaldehyde (**TMSPym-CHO**).

**Figure 3.7**: ^1^H-NMR (CDCl_3_, 400 MHz) of **ethinyl adamantane.**

**Figure 3.8**: ^13^C NMR (CDCl_3_, 400 MHz) of **ethinyl adamantane**.

**Figure 3.9**: ^1^H-NMR (CDCl_3_, 400 MHz) of 6-((adamantan-1-yl)ethynyl)nicotinaldehyde (**AdPyr-CHO**).

**Figure 3.10**: ^13^C-NMR (CDCl_3_, 400 MHz) of 6-((adamantan-1-yl)ethynyl)nicotinaldehyde (**AdPyr-CHO**).

**Figure 3.11**: ^1^H-NMR (CDCl_3_, 400 MHz) of 2-(Ethynyl-adamantyl)-pyrimidine-5-carbaldehyde (**AdPym-CHO**).

**Figure 3.12**: ^13^C-NMR (CDCl_3_, 400 MHz) of 2-(Ethynyl-adamantyl)-pyrimidine-5-carbaldehyde (**AdPym-CHO**).

# 4 References

1.	Maier, F. and O. Trapp, *Effects of the Stationary Phase and the Solvent on the Stereodynamics of biphep Ligands Quantified by Dynamic Three-Column HPLC.* Angewandte Chemie International Edition, 2012. **51**(12): p. 2985-2988.

2.	Trapp, O., et al., *In Situ Mass Spectrometric and Kinetic Investigations of Soai's Asymmetric Autocatalysis.* Chemistry – A European Journal, 2020. **26**(68): p. 15871-15880.

3.	Trapp, O., *Unified Equation for Access to Rate Constants of First-Order Reactions in Dynamic and On-Column Reaction Chromatography.* Analytical Chemistry, 2006. **78**(1): p. 189-198.

4.	Trapp, O., *A novel software tool for high throughput measurements of interconversion barriers: DCXplorer.* Journal of Chromatography B, 2008. **875**(1): p. 42-47.
